# Centering and collaborating with community knowledge systems: piloting a novel participatory modeling approach

**DOI:** 10.1186/s12939-023-01839-0

**Published:** 2023-03-13

**Authors:** Yahya Shaikh, Muzamillah Jeelani, Michael Christopher Gibbons, Denisa Livingston, David Rudyard Williams, Sanith Wijesinghe, Jenine Patterson, Sybil Russell

**Affiliations:** 1 The MITRE Corp, 2275 Rolling Run Dr, Windsor Mill, Woodlawn, MD 21244 USA; 2 International Islamic University of Malaysia, Jalan Gombak, 53100 Kuala Lumpur, Selangor Malaysia; 3 The Greystone Group, Inc, 9900 Greenbelt Rd, STE E103, Lanham, MD 20706 USA; 4 Strategic Ancestors, PO Box 235, Fruitland, NM 87416 USA; 5 Harvard University, 677 Huntington Avenue, Boston, MA 02115 USA; 6 The MITRE Corp, 7525 Colshire Dr, McLean, VA 22102 USA

**Keywords:** Participatory modeling, Community, Voice, Collaboration, Collaborative research, Equity, Disparities, Othering, Marginalization, Epistemic violence

## Abstract

**Background:**

Systems science approaches like simulation modeling can offer an opportunity for community voice to shape policies. In the episteme of many communities there are elders, leaders, and researchers who are seen as bearers of historic knowledge and can contextualize and interpret contemporary research using knowledge systems of the community. There is a need for a systematic methodology to collaborate with community Knowledge Bearers and Knowledge Interpreters. In this paper we report the results of piloting a systematic methodology for collaborating with a community Knowledge-Bearer and Knowledge-Interpreter to develop a conceptual model revealing the local-level influences and architecture of systems shaping community realities. The use case for this pilot is ‘persistent poverty’ in the United States, specifically within the inner-city African American community in Baltimore City.

**Methods:**

This pilot of a participatory modeling approach was conducted over a span of 7 sessions and included the following steps, each with an associated script:

- Step 1: Knowledge-Bearer and Knowledge-Interpreter recruitment
- Step 2: Relationship building
- Step 3: Session introduction, Vignette development & enrichment
- Step 4: Vignette analysis & constructing architecture of systems map
- Step 5: Augmenting architecture of systems map

**Results:**

Each step of the participatory modeling approach resulted in artifacts that were valuable for both the communities and the research effort.

Vignette construction resulted in narratives representing a spectrum of lived experiences, trajectories, and outcomes within a community. The collaborative analysis of vignettes yielded the Architecture of Systemic Factors map, that revealed how factors inter-relate to form a system in which lived experience of poverty occurs. A literature search provided an opportunity for the community to contextualize existing research about them using realities of lived experience.

**Conclusion:**

This methodology showed that a community Knowledge Bearer can function as communicators and interpreters of their community’s knowledge base, can develop coherent narratives of lived experiences within which research and knowledge is contextualized, and can collaboratively construct conceptual mappings necessary for simulation modeling. This participatory modeling approach showed that even if there already exists a vast body of research about a community, collaborating with community gives context to that research and brings together disparate findings within narratives of lived experience.

**Supplementary Information:**

The online version contains supplementary material available at 10.1186/s12939-023-01839-0.

## Background

### Representing the margins using knowledge systems of the centered – evidence-informed policy making?

Communities marginalized by systems of power can be further marginalized by being objects of research within those systems. Epistemic [1] violence [2–6] can occur along the knowledge production pipeline (i.e. funding; question framing and formulation; research paradigm selection; methodology, methods, and design; data collection; data cleaning, analysis, interpretation; publication and dissemination; impact and relationship to current knowledge, theories, policies, interventions; etc.) [3, 7, 8]. By the time a community on the periphery has been reduced to data and exits the knowledge production pipeline, reconstructed as an output of a centered system of knowledge, it may find its voice silenced, [2, 6] may not recognize its representation [9–13] and the knowledge production effort might be seen by the community as extractive to benefit researchers, institutions, and academic enterprises that uphold existing systems of power [14–18].

The challenge then is that while research and policymaking in the center may intend to benefit communities on the periphery, the dominant system of knowledge and understanding – the dominant episteme – through which that intention is operationalized is designed by the center and marginalizes the very people (and their marginalized epistemes) that they purport to benefit. Voice of marginalized communities (“epistemically disadvantaged identities”) [19] is silenced [2] within the dominant episteme through several mechanisms, including undervaluing or rejecting the speaker as a knower, [6, 20] and discrediting the knowledge and information from the Othered [21].

### Collaborating with knowledge systems from the margins through Fairness, Agency, Inclusion, and Representation (F.A.I.R.)

A considerable body of literature has pointed out that if knowledge is to have a transformative impact on society, there is a need for community participatory approaches to knowledge production (e.g. participatory action research, [22] mode-2 knowledge production, [23] transdisciplinary research, [24] civic science [25]). This includes the recognition that even highly “objective” fields of knowledge such as science and technology studies are culturally and politically situated [26–28] and that there is a need for co-production of scientific knowledge [29]. Similarly, a need for co-production is recognized in both over-arching spaces such as policymaking and public administration [30, 31] and in very specific challenges such as sustainable development and climate change [32, 33]. In general, literature points out that scientific expertise that lacks community perspective has proven insufficient for generating knowledge and policies that are high quality, socially relevant, robust and acceptable [29, 32, 33]. The importance of involving community voices and views takes on particular urgency with challenges of existential relevance such as climate change, [34–36] where there is a need for making societal demands and aspirations the basis for real solutions [37]. In the contemporary era of public governance, a more collaborative relationship is expected between state and civil society [30, 31] to collectively design and produce public goods and services, such as policies, urban services, disaster risk management strategies, and more [38–40]. Co-production has been adopted as a foundational aspects of some spaces such as sustainability sciences [38]. Yet, there are persistent gaps and challenges: current conceptualizations of co-production must go beyond stakeholder engagement by scientists to outputs that empower communities for social transformations and societal transitions; [41] they must re-engineer the institutional arrangements that govern relationships between the researcher and the researched and the governing and the governed; [38] and co-production and community collaboration is far from being mainstream [42]. Additionally, co-production and community collaboration does not automatically mean that the episteme of the community is represented in the effort. Co-production with a marginalized community can happen through dominant ontologies and epistemologies, which makes the collaboration more of a sense-making effort to translate experiences of the margin into conceptualizations for the center rather than an actual representation of the margins as they view and understand themselves [2, 6].

A number of strategies have been proposed to co-produce with communities in a way that overcomes epistemic violence in research and policymaking efforts [3]. These strategies can be summarized as prioritizing fairness, agency, inclusion, and representation (which can be summarized with the acronym FAIR) in the research or policy making effort [4, 7, 8, 21]. This includes acceptance of the speaker as *Knowledge-Bearer*, valuing their knowledge and information as an Othered voice, and recognizing their agency to use what they know to create new knowledge [2, 7–9, 20]. It also includes allowing community to be *Knowledge-Interpreters* to use their own ontologies and epistemologies to interpret knowledge and existing research about them so they can transform and contextualize it in a way that is recognizable to them as part of their own experience [2, 7–9, 20]. The role of Knowledge-Interpreter signifies someone who intimately understands the varied lives being lived in the present and the systems shaping those lives. Consistent with the designation of a “multi-disciplinary” researcher, [43] the role of a Knowledge-Interpreter (i.e. someone who is rooted in the episteme of their own community and has engaged in representing their community to another through advocacy or research about their own community) implies a “multi-epistemic” researcher. It requires a person to be rooted within the knowledge system of their own community while being able to translate and interpret that knowledge into another episteme (e.g. the centered episteme) to the extent possible, on and with their own terms. While a Knowledge-Bearer (e.g. an Elder who knows the lives of community members) and a Knowledge-Interpreter can be different individuals, both roles may also be found in the same person (e.g. a community leader who is also a researcher).

The strategy of collaborating with a Knowledge Interpreter is consistent with literature that suggests that knowledge useful for policy making is distributed amongst a spectrum of community members [44–46]. In fact, a criterion for identifying the Knowledge Interpreter is that they should have engaged with a broad spectrum of their own community as part of their research or advocacy work. The use of a Knowledge Interpreter additionally allows for the representation of communities for whom a normative role of a “Knowledge Bearer” exists as a part of their ontology, and their system of knowledge is informed, shaped, and represented by the individual(s) with a designated role of receiving, collecting, safe-guarding, and transmitting that community’s knowledge of itself [47–49]. By utilizing a Knowledge Interpreter the researcher removes themselves from being an interpretive lens, leaving community ontology and epistemology unencumbered to represent relationally and in harmony what external researchers can only perceive diametrically.

A number of approaches have evolved to address the need for community collaboration and co-production (e.g. community-based participatory research (CBPR), participatory action research (PAR), integrated knowledge translation (IAK), CBPR augmented by Human Centered Design (HCD)) [50–58]. Many of these methodologies attempt to undo the monopoly that centered institutions have on knowledge production and have shown to have significant success. However, there are persistent challenges that remain, many of which are related to researchers external to a community attempting to engage many members of that community, such as hesitation from community members; [57, 59] the complexity of relationship of outside researchers with local individuals hired to assist in the research process; [60] creating new or entrenching existing disparities in power structures; [61] logistic challenges of time and resources; [57, 59] the potential for unintended consequences when external researchers engage with community without having insights into historic and contemporary complexity of power structures; [61] and the possibility of failing to capture the heterogeneity of a community across its interrelated axes of differences [59].

The FAIR Framework attempts to address the “centered researcher/episteme in a marginalized community” problem by identifying multi-epistemic researchers (i.e. Knowledge Interpreters) as community members who are entrenched in the ontology and epistemology of their community, have already engaged in participatory works in their own communities, and are able to interpret sources of information from their community into the ontology and epistemology of the researcher external to the community. Through this process the community’s episteme and self-produced body of knowledge is centered and the external researcher’s role shifts to that of a student to be educated by a community’s existing interpreters and sources of knowledge.

### Systems science and simulation modeling as a means for self-representation for policymaking

A systems science approach rooted in community perspective is necessary to understand lived experience and the nonlinearity and emergent phenomenon (e.g. disparities, unintended consequences) intrinsic to the interaction between a policy ecosystem and community realities [62]. Specifically, approaches such as agent-based modeling (where a system is modeled as a collection of autonomous decision making entities [63]) are suited to simulate a system of individuals within communities whose interaction with policy ecosystems leads to emergent phenomena, some of which may be unintended consequences such as entrenching or creating disparities and poverty [63, 64]. Systems science is an interdisciplinary field focused on understanding the inter-relations and interactions between entities that comprise and function as a whole. Simulation modeling is a method used in systems science to develop a simplified representation of reality, often used to better understand and anticipate behaviors of the real-life system. Constructing a simulation model to inform policy making requires the recognition that policies are not implemented in physics-based systems with well characterized causes and effects, but rather policy always works within socio-technical systems [65]. To understand these systems there is a recognized need for better representation of the cultural, economic, and social aspects that are influenced by and are influencing politics [65]. Constructing well-formed policy simulation models would require intimate knowledge of lived-experience from communities. However, because many researchers doing simulation modeling are often not from the communities they are modeling, it is recommended that modeling be done in collaboration with communities [66]. Collaborating with communities to develop a model allows for representation of local realities into the simulation, increases social capital of communities, and increases probability that the model may successfully influence decision making [67]. While collaboration allows communities to shape a process, they themselves get shaped in return: new relationships often form that leads to social capital increasing in the participatory process [68].

The closely related discipline of computational social science predominantly uses agent-based methods, in which software agents are used to model and simulate individuals and resulting populations. By providing rules that are applied by the individuals based on their current perception of their situated environment on the micro level, the behavior of the population on the macro level is generated, which again can lead to emergent behavior. Examples for this approach are focused approaches on pathways out of poverty, [69] but also more general evaluation frameworks, such as provided from a systems perspective [70]. A general overview of the state of the art was compiled by Gilbert et al. [71]. The agent-based metaphor also allows for the application of participatory methods, as different groups can define rules and behavior of agents representing their worldview and perceptions. Examples are given in the recent compendium on Human Simulation [72]. Agent based models and the system science approach are mutually supportive methods and often combined in hybrid modeling approaches supporting cross-disciplinary views of common challenges. A collaborative simulation modeling effort can function to represent the factors and relationships that shape a community’s reality through their perspective. Outputs of a collaboration based on F.A.I.R. can in turn serve to inform policymaker’s efforts.

### Piloting A F.A.I.R. approach to collaboration – low socioeconomic status as a use case

In this paper we report the results of piloting a systematic methodology for collaborating with a community Knowledge-Bearer and Knowledge-Interpreter to drive the development of a simulation model. The use case (or exploratory space) for this pilot is that of ‘persistent poverty’ in the United States, specifically within the inner-city African American community in Baltimore City. The goal is for the community Knowledge-Bearer and Knowledge-Interpreter to generate an architecture of systems map that can be used as an input to construct a simulation model of poverty. The purpose of the architecture of systems map is to reveal factors and their relationships as an exploration of the community’s categorization of ‘persistent poverty’.

How poverty is defined and understood has profound implications. It informs who is designated as “poor”, how policies are shaped, which communities get which resources, how success is measured, and what next steps should be [73]. The current income-based definitions of poverty used by the federal government had early foundations in the 1955 USDA food consumption survey, which revealed that families of three or more spent about one-third of their income on food [74]. This led to an income-based definition of poverty that persists till now (with annual adjustments of the poverty income thresholds to reflect changes in the cost of living) and which informs community designations, classification of discrete populations, including the identification of "persistently poor counties" (PPCs) as counties that have had 20 percent or more of their population living in poverty over the past 30 years [75]. These designations shape policy interventions and targeting of resources such as the introduction of the 10–20-30 plan as part of the *American Recovery and Reinvestment Act of 2009* to direct relevant federal programs to direct at least 10% of total investments to PPCs [76]. The *Combating Persistent Poverty, 10–20-30 Works* report highlighted the success of the 10–20-30 formula and suggested its expansion as a next step, [77] with actual expansion enacted under the *Targeting Resources to Communities in Need Act of 2022* [75]. In the US and in other countries, national conversations about poverty are often rooted in an income-based, poverty-line approach [78, 79]. Because of the wide-ranging impact stemming from how poverty is understood, assessing and enriching that understanding becomes critical. The etiology, impact, and experience of poverty differs for the many communities that make up the US [74] – from the Colonias of the Rio Grande to the counties of Central Appalachia to Indigenous communities and inner cities. Basing conversations primarily on income means they are disconnected from the lived experience of poverty, the reality of people’s lives, and their living conditions [78, 79]. Scholars studying poverty have pointed to the need for an approach rooted in community realities to understand how policies and interventions achieve varying levels of success or create unintended consequences such as exacerbating disparities [80]. For example, policies that attempt to reduce fraud in public benefit programs can increase the complexity of the application and in turn become barriers to access the benefits or lead to disqualification of a person for making an error on a complicated application [80]. Another example are policies that seemingly benefit everyone, but in fact end up producing greater benefit in those communities represented by the policymakers because other voices and realities were missing at the time of the policymaking process [81]. At times policymaking and intervention design occur at a far enough distance from community realities that they produce the opposite result of their intent. The World Bank Report, “Voices of the Poor: Can Anyone Hear Us?” recounts the collision of a poverty alleviation intervention with community realities [82]:

In Philippines, in the Mindanao region, women said “we boil bananas for our children if food is not available. In some cases, when the Department of Agriculture distributes corn seeds, we cook these seeds instead of planting them.” Ironically, they borrow money to acquire these seeds. The cycle of poverty continues as they are unable to pay for these loans.

Of note, ‘community realities’ include both negative factors that shape communities (such as historic traumas and contemporary policies) and positive factors intrinsic to that community (such as social networks and community-based organizations that allow it to survive and thrive despite disadvantaging systems).

### Community voice in agent-based modeling of low socioeconomic status – current solutions and gaps

Many studies that have developed agent-based models related to poverty point to the need to incorporate further information as next steps to effectively represent the system shaping poverty, [83] much of which can be gathered through community input and collaboration. However, there are a number of unique challenges related to incorporating community realities into simulation and modeling of policies. Community voice as a result of qualitative data collection may require a sample size that may not be realistic for policymaking efforts. For example, in the United States, limitations to engaging a sufficient sample size in a timely way may be rooted in legislation such as the Paperwork Reduction Act, which imposes procedural requirements on agencies wishing to collect information from the public [84]. These requirements, including obtaining approval from the Office of Management and Budget (OMB) before collecting information from ten or more respondents outside the federal government, effectively limit the extent to which policymakers may engage with communities in an agile manner [84]. Yet, to make effective policies it is necessary for community voice to represent the architecture of factors shaping its realities.

Additionally, incorporating community voice for policy simulation means being able to explore how community experience including marginalization is constructed through a variety of policies. Participatory modeling scripts allow for the exploration of experiences to construct conceptual models [85]. A survey of scripts reveals that there is an availability of scripts for participatory modeling of policies, often for systems dynamics models [86, 87]. However there is a lack of scripts related to exploring the connection between community experiences including marginalization and policies for agent-based models.

Furthermore, because communities thrive even under considerable marginalization, knowing a community's assets in addition to barriers can help determine the shape of policies. While many scripts capture the system around a community, there is a lack of scripts with an intentional focus of identifying and describing assets in addition to barriers.

In trying to represent community voice, communities often point to individuals from amongst themselves that are bearers of knowledge, tradition, and are a part of the system of reality in that community. In many cultures such as in indigenous and African-American communities, an elder or community advocate or community researcher from that community often fulfills this role of Knowledge-Bearer, interpreter, and transmitter. However, simulation models have utilized the modeler to interpret research about a community or have utilized the modeler’s own knowledge base to construct models [69, 83]. Specifically related to poverty, in constructing simulation models, researchers have often utilized quantitative data like household surveys, [88] theory, [83] field studies, [69] and a combination of theory and a modeler’s own expertise [69, 70, 83]. These methodologies are often limited by a modeler from outside the community having to interpret surveys, theories, and field studies, with a resulting lack of generalizability of findings or even bias against marginalized populations [89–93]. To the best of our knowledge, there has been limited work to develop and formalize participatory methods to use a community’s Knowledge-Bearer and Knowledge-Interpreter to build agent-based models for policy development. Fortunately, many communities have elders, advocates, and researchers from their own membership that have generational knowledge and wisdom; have engaged in understanding and communicating the lived experiences of their community; or may have conducted numerous interviews, focus groups, surveys, questionnaires, and research on their own community. By leveraging a community's Knowledge-Bearer – an elder or advocates or researcher – we can center rather than marginalize a community's knowledge production and epistemology. Having a Knowledge-Bearer indigenous to a community who can interpret the body of research and lived experiences means we may be able to: utilize a community’s own knowledge and research in synthesizing hundreds of voices from their communities; leverage their research efforts over many years within their own community, which may far exceed what is possible or, for government, legislatively permitted in a single research effort; and, in comparison to strategies that utilize a modeler's knowledge base, has the potential to have a more accurate representation of a community's lived experience.

There is a need for participatory methods that privileges community interpretations of themselves, and which engages the medium of a Knowledge-Bearer (e.g. elder, advocate, researcher) from a given community to explore that community’s research and representations. Existing methods for participatory modeling scripts often seek to engage with community members, but with sample sizes which may be onerous in efforts relating to policymaking (e.g. a survey by federal policy makers in the US involving 10 or more participants would require undertaking an OMB approval process); [94] and are often not directly applicable in scales of analysis outside of systems-dynamic modeling, such as for agent-based models [67].

To address this challenge, we developed and piloted a systematic methodology consisting of novel scripts to collaborate with a community’s Knowledge-Bearer and Interpreter as a medium for representation and interpretation of a community’s knowledge of themselves; and to explore those interpretations systematically in order to construct artefacts relevant to developing simulation models to support policymaking. In this paper, we describe the process and outcomes from piloting this novel participatory model building approach in relation to an initial use case of persistent poverty.

## Methods

This study utilized subject matter experts, did not engage in human subjects research, and was exempt from IRB review.

### Design, timeframe, and setting

This pilot of a participatory modeling approach was conducted over a span of 7 days and included the following steps conducted over 7 sessions:

- Step 1: Knowledge-Bearers and Knowledge-Interpreter recruitment
- Step 2: Relationship building
- Step 3: Session introduction, Vignette development & enrichment
- Step 4: Vignette analysis & constructing architecture of systems map
- Step 5: Augmenting architecture of systems map

This analysis was focused on exploring the experience of poverty in communities experiencing “persistent poverty”. Persistent poverty communities are those where 20% or more of the population has been below the federal poverty line for the last 30 years or more [75]. These communities are broadly clustered together with contiguous counties forming discrete regions in central Appalachia, in the Black Belt, along the Mississippi River and delta, and along the US-Mexico border including the Rio Grande Valley. A non-contiguous group of communities form the Native Nations “cluster” and, separately, the inner-city African-American community “clusters”. Due to recent events and media focus highlighting structural violence against African-American communities (e.g. police brutality and disparities in COVID-19 mortality), we selected the inner-city communities of Baltimore city to pilot this participatory methodology.

The discrete steps in this participatory methodology each have an associated script and outputs, which form the inputs for the next step (Table 1) and are detailed below.

**Table 1:** Steps of the FAIR Framework for participatory modeling

Step	Script(s)	Output
Knowledge-Interpreter Recruitment		Community members; Knowledge-Interpreters
Relationship Building		Community knowledge interpretation & collaboration
Session introduction, vignette eliciting & development	Narrative / Vignette Eliciting & Development Script	Vignettes / narratives
Vignette Enrichment	Narrative / Vignette Enrichment Script	Enriched Vignettes / narratives
Vignette Analysis & Constructing Architecture Of Systems Map	Narrative / Vignette Analysis ScriptArchitecture of Systems Conceptual Model Building Script	Architectures of Systems Map
Augmenting architecture of systems map	Literature Review	Augmented Architectures of Systems Map

### Knowledge-bearer / knowledge-interpreter recruitment

In attempting to root research paradigms within a community it needs to be recognized that there already exists a fund of knowledge, research, and analytic capacity about that community within that community. At times a “knowing” that a community has about itself is left unaffirmed or even contradicted by external institutions and knowledge [95]. In situating this study within a “knowing” that a community has about itself (e.g. knowledge transmitted across generations; research carried out by members of a community), we engage with an elder, advocate, or researcher from that community as “Knowledge-Bearer”. We define “Knowledge-Bearer” as someone who carries knowledge about their community and lives in relation to them and that knowledge. An example is a community elder or a community leader who lives within their community, understands the diversity of experiences and systems of realities, and lives in relation to that knowledge through leadership, activism, or advocacy. A “Knowledge-Interpreter” is a community member who is rooted in the episteme of their own community and who have previously conducted research, focus group sessions, interviews, or advocacy work directly and peripherally related to the topic of the study as part of their professional and community work. The Knowledge-Interpreter is rooted within their own community’s episteme and able to represent their community’s reality into another episteme (e.g. the centered episteme). A Knowledge-Interpreter unifies the concept of “researcher” and “researched” within a single body. While the community being researched forms the subject of a Knowledge-Interpreter’s professional and community work, they are also the object of their own inquiry and self-representing through their lived experience within their communities. In addition to interpreting community experience into another episteme, a Knowledge-Interpreter would have the capacity to interpret, contextualize and make coherent otherwise disjointed and fragmented literature, narratives, and research about the community. A Knowledge-Bearer and a Knowledge Interpreter can be distinct people or both roles can be the unified within the same person if a Knowledge-Bearer, speaking to the lived experiences of a community, is also engaged in representing their community through research and advocacy. The Knowledge-Bearer / Knowledge-Interpreter would function as a subject matter expert to create vignettes and engage in participatory modeling.

An internet search was conducted to identify potential Knowledge-Bearers / Knowledge-Interpreters in Baltimore City. The following inclusion criteria were used to optimize the probability that the Knowledge-Bearer / Knowledge-Interpreter identified for participation has knowledge of a diversity of experiences within their community, is considered by their community as a leader that can represent them, and can speak to the space being examined (i.e. poverty): [96, 97].

- (1) Should have engaged in efforts within their community that included discussions with key informants, focus groups, and a diversity of voices and lived experiences
- (2) Hold formal positions in the community that is the focus of this study
- (3) Have knowledge relevant to the study, be willing to share this knowledge, and communicate well
- (4) Be unbiased or able to reflect upon their own biases
- (5) Due to timeline constraints, they should also be immediately available and easily accessible

The capacity for reflexivity [98] was demonstrated by discussions about their past work in regards to: the extent to which they included a wide range of different perspectives within their work, the impact of their own background on how members of their own community interacted with them and influenced the outcomes of their work, and the psychological impact of doing their work on participants. As a result of our search we identified a Knowledge-Bearer / Knowledge-Interpreter for inner city Baltimore who was previously an associate director of an institute based at a large academic center in the city and focused on the health of urban communities. As part of his work spanning several decades, he has conducted hundreds of focus group, and key informant and personal interviews across a wide spectrum of community contexts and outcomes. He is a recognized national leader representing the voices of inner-city communities to inform state and federal agency initiatives.

### Relationship building

The exploration of a community's knowledge about themselves, whether it's quantitative or qualitative data collection, constitutes intimate knowledge. Research on vulnerable and marginalized communities has at times been an extractive enterprise, serving to further academic careers [11–13] and corporate profits [17, 99]. Even worse, it has at times served to entrench stereotypical and marginalizing representations [10, 16] and, sometimes even led to the violation of the sacred space by dismissing or refuting community ontologies and epistemologies [10, 18, 100].

For communities, trust and faith consecrates interactions, transmogrifying them into relationships. It’s within that sacred relationship-space that research or information gathering for policymaking can occur. In the flow of insight and information from community to an external audience an assumption of trust and a leap of faith is made by community that the information they shared would be used in some positive way. There is a trust that it would not be used in extractive or disenfranchising ways. There is blind faith in the connection formed with the researcher or policymaker that thoughts, insights, and information they have entrusted is not going to be weaponized against them in ways that are othering or challenging and negating of identities, ontologies, or epistemologies of the community. The space in which community entrusts a part of themselves to another becomes sanctified and sacred by virtue of the trust, faith, and transcendence involved in the giving. When what is given is used by researchers or policymakers in ways that lack fidelity to the expectation of the giver during the meeting in the sacred space, then the researchers’ actions are a betrayal of faith, trust, and desecration of what was sacred [10].

In order for a community to have some surety that the sacredness of the community’s sharing will be safeguarded and to ensure a collaborative exploration, Knowledge-Interpreters need to be comfortable with the intentions, approaches, and goals of the effort [101]. Even if well-intentioned, such explorations can serve to re-traumatize communities so the processes and outputs of the effort need to have a net effect of being empowering to the community: they need to be liberating in some way and could be used by the community towards their own ends. The intentions and goals of this research were discussed over several conversations to give the Knowledge-Interpreter an opportunity to ask questions, space to reflect, and an ongoing opportunity to disengage if desired. From the outset this effort was intended to be helpful to communities in highlighting their intrinsic assets and resilience in the face of factors architected to shape their realities. Additionally, agent-based simulation constructed from the output of this effort would be made freely available to communities for their own purposes (e.g., advocacy).

### Session introduction, vignette development and enrichment

Vignette development and enrichment spanned two sessions. In the initial session participants (i.e. Knowledge-Bearers) were introduced to the topic of persistent poverty and the participatory methods being utilized. At the end of the session the Knowledge-Interpreter was asked to recall their experiences they had in interacting, collaborating with, and representing the diverse voices within their communities. Based on these recollections they were asked to write vignettes relating to the following prompt (see details in the Narrative / Vignette Eliciting & Development Script):

Keeping in mind the diversity of people and experiences in your community, write up to 5 vignettes. Each vignette should describe the lived experience of 'poverty' of a fictional individual that can be a composite or anonymous case representing a sub-segment of the community.

During vignette enrichment the Knowledge-Interpreter explored and reflected on each vignette. These explorations sought to build each vignette into a richer narrative and to help identify how discrete factors related to each other. The facilitator used a script that was informed by the muti-dimensional conceptualization of poverty and the relationship between elements that lead from nascent causes to community level outcomes.

**Fig. 1:**
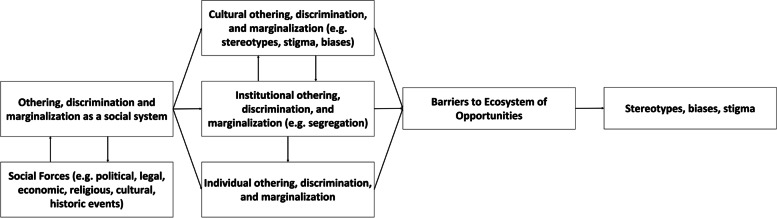
Abstracted Williams-Mohammed Framework

The multi-dimensional conceptualization of poverty recognizes that economic and non-economic dimensions of people’s lives are affected by poverty and that poverty occurs within and is impacted by political, economic, social, and cultural contexts [73]. An abstracted version of the Williams-Mohammed Framework for the Study of Racism and Health, which begins by examining social forces and marginalization as contextualizing factors, was used to systematically uncover the presence of disadvantaging factors and relationships shaping the vignette. The Williams-Mohammed Framework was selected for its demonstrated validity in the field, it's multi-dimensional approach that allows it to be applied across communities, and its completeness in relating othering to outcomes [102–104]. This Framework was generalized for application beyond racism and health by abstracting racism as marginalization and discrimination, and references to health and its social determinants as access to opportunities [102–104]. The abstracted Williams-Mohammed Framework (Fig. 1) was used as a template for generating questions to explore relationships between factors salient in the narrative and related (i.e. preceding and succeeding) factors along the pathway from structural elements such as policies and marginalization to outcomes such as poverty.

**Fig. 2:**
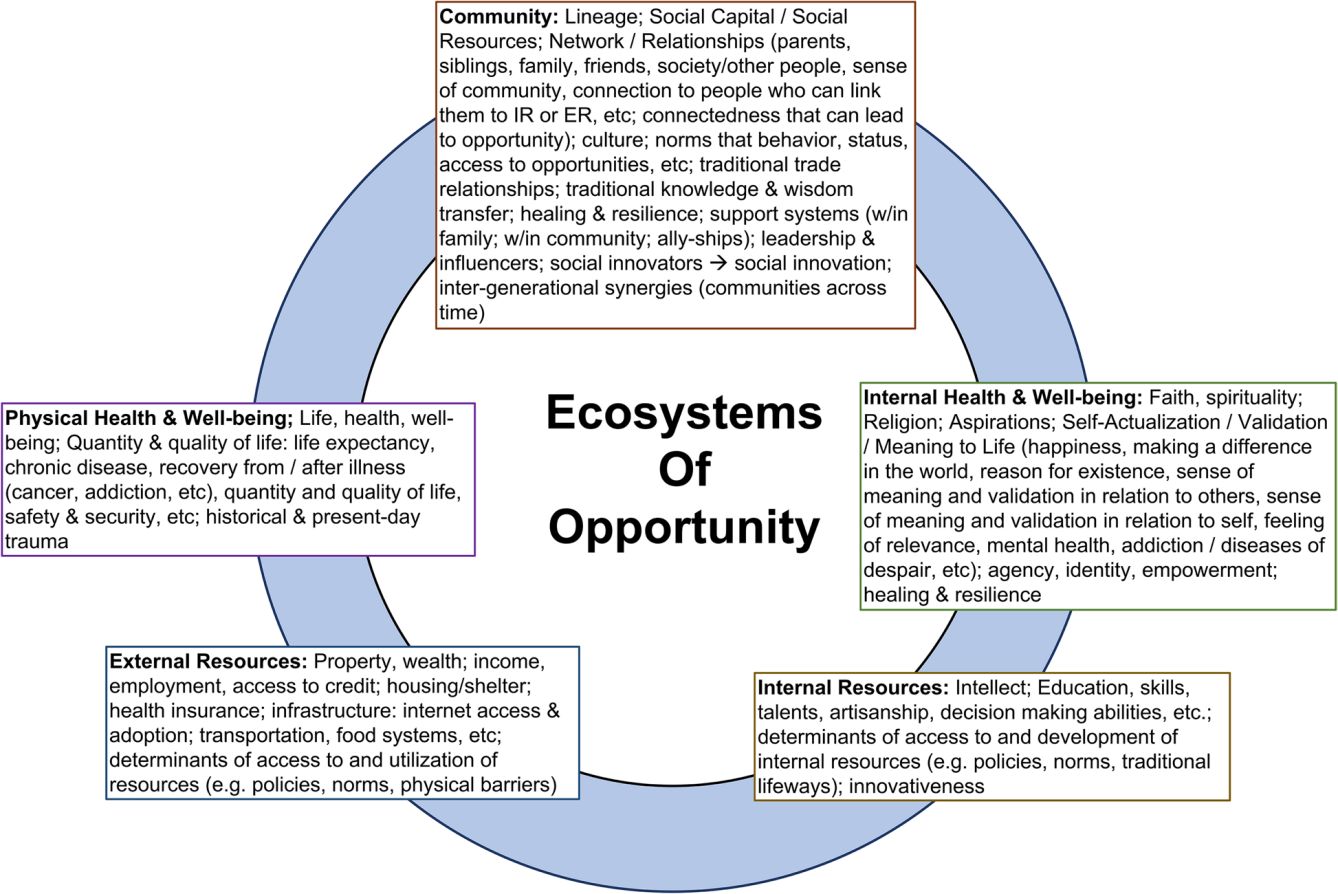
Ecosystems of Opportunities meta-model

Studies that use the voice of communities to explore poverty [82, 105, 106] often converge on a handful of domains to characterize the multi-dimensionality of poverty [73]. These domains form an ecosystem of factors that occur in various permutations for different communities to shape their experience of poverty and access to opportunity [73]. These domains were reviewed by previous studies [73] and are summarized by us in the Ecosystems of Opportunity meta-model presented in Fig. 2. The Ecosystems of Opportunities meta-model, which categorizes community resources into five domains, served as an open-ended exploration of assets and their relationships that exist within the community. Combining the Williams-Mohammed Framework with the Ecosystem of Opportunities meta-model allowed for a representation of both community assets and deficits (See Narrative / Vignette Analysis Script).

### Vignette analysis and constructing architecture of systems map

Vignette analysis spanned a series of 3 sessions. During these sessions analysis of vignettes were conducted according to Glaser and Strauss's [107] grounded theory approach. Following the guidelines set out by Strauss, [108] open, axial and selective coding of the vignettes were done in collaboration with the participating community’s Knowledge-Interpreter. Extensive memoing was done to elaborate code categories and to conceptually relate codes with each other. Important concepts were discovered using concept respecification, [109, 110] leading to the development of a concept-indicator model [109–111] which, in its visual representation, evolved to become the architecture of systems map. Analysis was done in collaboration with the Knowledge-Interpreter and note-taking and map construction was done by the session facilitator (See Architecture of Systems Conceptual Model Building Script).

### Augmenting architecture of systems map

After completing vignette enrichment, analysis, and construction of an architecture of systems map, each linked pair of concepts in the map formed search terms that were used to conduct a literature search using Google Scholar. When a relationship was identified in literature, the citation was added to the map, and where necessary the map was augmented with any additional factors and relationships (See Architecture of Systems Conceptual Model Augmenting Script).

A web-browser based application was developed to simplify the process of implementing the methodology above including data collection for vignette development, diagramming and augmenting the architecture of systems map, conversion of diagram into a computable JSON object, and subsequent annotation for agent-based modeling.

### Session evaluation

After each session, the Knowledge-Interpreter was asked if the sessions were useful, if they uncovered details that were insightful, and whether the sessions should be improved in any way. Because an exploration of architectural factors shaping community realities discusses historic and ongoing traumas on the community, the discussions themselves can serve to re-traumatize. There was space given after each session to discuss the format, spreading out discussions in order to give time to heal; prioritizing consent to engage and disengage to the extent they are comfortable, ensuring safety of the discussion space; ensuring transparency; and ensuring an ongoing relationship as collaborators leading an exploration of their own realities rather than objects of study.

## Results

Each of the activities—vignette development, vignette analysis and construction of architecture of systems map, and augmenting architecture of systems map—resulted in artifacts that were valuable for both the communities and the research effort.

### Vignette development and enrichment

Vignette construction resulted in a number of narratives that represented a spectrum of experiences, trajectories, and outcomes within a community. They yielded a narrative representation of lived experiences that summarized factors and their inter-relationships surrounding a community's reality. These narratives additionally gave space for communities to represent an asset-based perspective by pointing to a community’s aspirations, strengths, actualization of their worldview, and the creation of a unique space emergent from a network of relationships between a community of people in commune with the universe. These narratives allowed for an expression of a sense of self and a focus that extended beyond barriers or deficit-focused strengths such as ‘resilience’.

### Vignette analysis and constructing architecture of systems map

The collaborative analysis of vignettes yielded an initial architecture of systemic factors map, which was insightful for the objective of this study to better understand the factors that inter-relate to form a system in which lived experience of poverty occurs. At the same time it was enlightening to the community in visualizing how factors beyond their individual efforts and choices shape their lives and their outcomes. This artifact showed: how the past shapes the present (e.g. the connection between red-lining policies from the past and current neighborhood features such as employment and schools); the relationship between how factors that serve to advantage one community can be the very same factors that disadvantage another (e.g. how infrastructure is developed in a city that decreases the property value of some communities while increasing it for others); that policies that are seemingly advantageous to all can encode advantaged identities and be marginalizing to populations that don't share those identities, that a confluence of factors shape advantage and disadvantage for a community (e.g. how wealth accumulating behavior of White communities is encoded into the tax code, disadvantaging Black identities); that communities contain within themselves factors and assets that allows them to survive and thrive in systems that may be otherwise disadvantaging for them (e.g. a sense of community in a neighborhood such that children have multiple households that are nurturing spaces and many adults that are a source of mentorship for children); and that across different communities there are pathways that are conserved and also that differ while leading to the same outcome (e.g. a conserved pathway of a feeling powerlessness when experiencing discrimination and being left out of societal advantages).

### Augmenting architecture of systems map

**Fig. 3:**
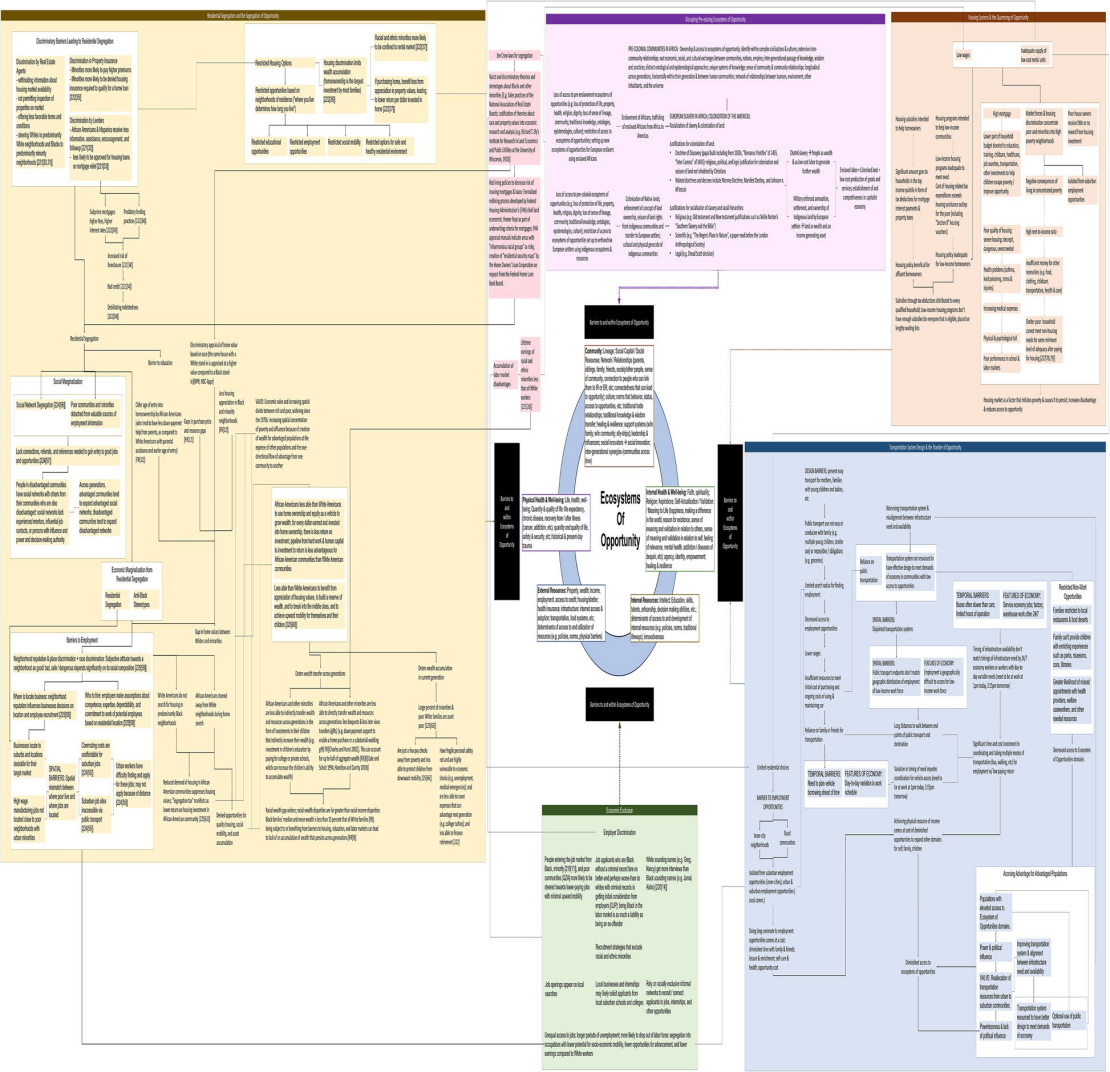
Architecture of systems map

A literature search examining each of the relationships surfaced in the architecture of systems map provided an opportunity for the community to contextualize existing research about them and hang published research about them on the frame of lived experience. It served to provide further insights into the system surrounding community realities. Some of the relationships were also recorded within literature, while other relationships that emerged from narratives were magnified further in literature through intermediary steps. The laborious process of conducting a literature search to examine relationships that emerged from community narratives was an exceedingly powerful process: it revealed the fracturing and fragmentation of community realities spread out across academic peer-reviewed siloes. This step led to the final Architecture of Systems Map (Fig. 3) and revealed the importance of community experience as the organizing force to inter-relate, synthesize, and reconstruct a coherent picture of the community that is otherwise spread disjointedly across a vast landscape of academic literature.

### Session evaluations

Results from post-session discussions revealed the value of the process to communities. Specifically, participants found that: (1) narrative exploration through open ended questions helped clarify how a confluence of historic, social, and policy factors restrict and shape choices and consequences within communities; (2) narrative exploration also helped to bring to light how strengths and assets in the community that allow it to survive and thrive against challenges that emerge from systems surrounding the community; (3) others in the community might benefit from seeing the ASF map so that they see how adverse outcomes in the community is not a consequence of an intrinsic defect within the community; (4) it was valuable to have engaged in an effort to develop a way to encode community voice and narratives into quantitative efforts like simulation modeling; and (5) it was valuable to have listened to narratives from a different community and seen the similarities and differences in how a common lived experience is shaped.

In summary, this process was a significant benefit in showing that even if there is a vast body of research about a community, there is an alternative to modelers and researchers engaging with that corpus by themselves. This methodology showed that indigenous researchers are effective interpreters of their community’s knowledge base, can develop coherent narratives of lived experiences within which research and knowledge is contextualized, and can collaboratively construct conceptual mappings necessary for simulation modeling.

## Discussion

We implemented an innovative strategy to engage with a community’s research base through collaborating with indigenous researchers. It showed a way to capture community representations, revealing the architecture of factors that form the system in which community lives are shaped, identifying policy determinants of community realities, and co-developing the structure and building blocks of an agent-based model.

We found that community developed vignettes formed a fertile foundation for an in-depth exploration of underlying factors and their inter-relationships. The vignettes were composites representing a segment of community population, with multiple vignettes serving to capture a diversity of stories within a single community. The multiple vignettes revealing a spectrum of trajectories and outcomes served as a counter to the tendency to fix and essentialize community identities [112–114]. The modification of the script with a known cause-effect structure such as the Williams-Mohammed Framework allowed an unstructured but guided exploration to identify factors and their relationships shaping a community’s lives. Additionally, it revealed a community’s perspective on mechanisms through which barriers to opportunity are created. Utilizing the Ecosystems of Opportunity meta-model was helpful in identifying assets that define a community and their relationship with the universe around them. The systems of architecture map created from community narratives and its augmentation with literature search creates a powerful dialectic between community and academia. The map provides a relational deconstructed visualization of factors that shape a community’s lived experiences, while a community’s lived experiences shapes and unifies fragmented studies into a coherent whole. This suggested to us the potential role of community experience as a methodology to drive literature reviews to re-construct a coherent picture from many different research efforts.

The architecture of systems output by this methodology is supported by literature that examines structural inequities [62, 115–123]. The architecture offers a useful way to bring together a wide spectrum of studies into a cohesive narrative whose construction emerges from community Knowledge-Bearers.

The F.A.I.R. methodology is timely in light of the *Executive Order On Advancing Racial Equity and Support for Underserved Communities Through the Federal Government*, which stated “because advancing equity requires a systematic approach to embedding fairness in decision-making processes, executive departments and agencies (agencies) must recognize and work to redress inequities in their policies and programs that serve as barriers to equal opportunity” [124]. The EO tasked each agency to conduct a review of select policies and programs, to which they responded with recommendations specific to their agency. However, the results of capturing community voice through the FAIR Framework revealed communities don’t experience policies as discrete individual impacts. Rather, in the lived experience of communities interacting sets of policies from a spectrum of agencies form a “policy ecosystem”. The “policy ecosystem” interacts with community realities, in the process shaping individual choices and lives, and creating barriers and exacerbating disparities [125].

The methodology highlighted in this paper offers policy makers a systematic process to collaborate with communities, to give voice to their realities, and help map out systems of barriers. Mapping the architecture of systems impacting the community can lead to a simulation to anticipate how policies can interact with each other in a policy ecosystem and how, when applied to a community’s realities, may impact outcomes and disparities. Furthermore, this effort revealed an alternative to modelers and researchers interpreting research about a community. This effort showed a systematic methodology to collaborate with indigenous researchers as access points for a community's body of research and as Knowledge-Interpreters to inform model construction, to reveal the multi-dimensionality of a community’s reality, and to link those realities to policies. These types of insights can inform the modifiable inputs and visualizable outputs of an interactive simulation model. Furthermore, this effort underlines a significant space for Knowledge Interpreters – multi-epistemic researchers – as community researchers and advocates who straddle multiple epistemes. In the language of multiple vantage points contributing to knowledge production, [43, 126, 127]. Interpreters can be more accurately described as multi-epistemic researchers, who are urgently needed in efforts to recenter and diversify systems of knowledge.

Constructing an underlying conceptual model based on a community's knowledge base offers an opportunity to represent the perception of agents about their environment, and their interactions within it. By formally characterizing a community's perception of elements and relationships that make up the system within which they live their lives, a model can be enriched by agents' belief structures and perceptions that underly interactions and behaviors [128]. Self-characterization as done in this framework revealed a number of policies that shape community realities and can therefore be useful for policy simulation efforts [129, 130].

A limitation of this process was the emotional impact it has on community partners. The process of exploring systemic barriers had the potential to re-traumatize community participants. This was mitigated through ensuring that partners had the power and choice to stop or take breaks or reconvene at a later time. Additionally, detailed discussions at the end of each session offered an opportunity to discuss the emotional impact of the process and allowed voice to be given to feelings that were elicited. This methodology is also limited in that it in its representation of community experiences through narratives and conceptual models, it excludes epistemologies that have alternate ways of phenomenological expression such as through experience, performance, dance, production of tapestries, etc. To work with alternate epistemologies, an additional step at the beginning of the FAIR Framework would surface and conform subsequent steps to the community’s modes of knowledge generation, expression, representation, transmission, and recording.

A key lesson we learned in implementing the FAIR Framework is that relationship building is the most important and foundational step of the entire process. Establishing a relationship of trust meant that community researchers were driving priorities, direction, and exploration and felt comfortable enough to allow FAIR facilitators into the sacred space of their memories and experiences. There are a number of ways to monitor and shape the relationship building process for trust [101]. The formation of relationships around research efforts has the potential to elicit memories of past betrayals and traumas a community may have experienced [10–13, 16–18]. Relationship building requires investment to ensure there is space for healing, that benefit accrues to the community and isn’t extractive, and ensures community has control during the effort and ongoing control over any outputs even after the initial participatory effort is complete.

This effort is situated within academic knowledge-production whose output includes an audience of researchers and policymakers. A limitation of this situatedness is the knowing of the community’s Knowledge-Bearer that the results of this collaboration in the margins would be presented to an audience in the center through an academic journal. The fear is that by representing challenges the community faces it may serve to ingrain stereotypes even when surrounded by a visual model revealing an architecture of systems creating those challenges. Dotson refers to this fear as “testimonial smothering” and defines it as “truncating of one’s own testimony in order to insure that the testimony contains only content for which one’s audience demonstrates testimonial competence” [6]. Testimonial smothering is a mechanism of epistemic violence and is the consequence of the speaker’s and their community’s past experiences with the centered episteme and dominant systems of knowledge production, and their consequent anticipation that the testimonial may serve to further entrench misperceptions from the center about the margins [6]. To mitigate this concern, we intend to submit these findings to academic forums possessing an audience of communities on the margins: the Global South, post-colonial societies, and indigenous communities.

As next steps, we intend to convert the mappings into computational representations, which can be used in dashboards to help visualize the reason behind distributions of quantitative measures seen in data visualizations. We intend to discuss these mappings with community to identify and prioritize policy interventions. By translating these maps and policy strategies into agent-based models and policy levers, we hope to strengthen advocacy efforts and inform better policy making. Additionally, in this study we examined collaboration with a community’s Knowledge-Bearer / Knowledge-Interpreter to represent primary knowledge of lived experiences and also to reclaim and interpret academic literature about that community into a coherent self-narrative. In future research we hope to use the framework reported in this study to collaborate with community Knowledge-Bearers and Knowledge-Interpreters (i.e. multi-epistemic researchers rooted in the margins) on primary data collection efforts.

In conclusion, through this work we have added to the body of knowledge of participatory modeling of policies. We have contributed a methodology to: systematically collaborate with community Knowledge-Bearers / Knowledge-Interpreters to access and interpret a large body of community knowledge and voices; to reveal the impact of policy ecosystems on communities; and to surface equity considerations relevant to policymaking. Our work is promising for efforts that want to collaborate with communities for in-depth mapping of lived experiences to reveal policies shaping community realities.

## Supplementary Information

**Additional file 1.** Participatory modeling scripts.

**Additional file 2.** Output of participatory modeling scripts.

## Data Availability

All data (i.e. the narratives / vignettes) generated or analyzed during this study are included in this published article within the supplementary files.
