# Supplementary material for: Centering and collaborating with community knowledge systems: piloting a novel participatory modeling approach
Source: Int J Equity Health. 2023 Mar 13;22:45. doi: 10.1186/s12939-023-01839-0 (PMC10010640; doi:10.1186/s12939-023-01839-0)
Supplement: Supplementary file 1 — Additional file 1. Participatory modeling scripts. [file 12939_2023_1839_MOESM1_ESM.pdf]

SUPPLEMENTAL MATERIAL 1:

PARTICIPATORY MODELING SCRIPTS

NARRATIVE / VIGNETTE ELICITING & DEVELOPMENT SCRIPT:

Context: At the beginning of narrative collection or vignette development process

Purpose: Provide a platform and space for recording community voice and lived experience.

Time required during session: 2-3 hours

Materials needed:

- Voice recorder
- Laptop
- Notebook or blank paper
- Pens or pencils

Inputs:

- Diversity of lived experiences in community

Outputs:

- Series of vignettes or narratives

Roles: Facilitator to introduce session and process; Community member or key informants to voice lived experience

Steps: Each of the following can be adapted to match local vocabulary and language of the community. The facilitator introduces the purpose of the activity and the process of developing a vignette. The following may be one way to do an introduction:

- INTRODUCING THE PROJECT: This project hopes to understand “poverty”. Our part of the project is about hearing about and recoding your life and experiences. We call your story your “voice”. Your “voice” is your reality and includes the context of your story: what are the things before your life and during your life that made your story the way it is? Your “voice” also means how you interpret your story and what your story means. Your “voice” also means how you want your story to affect this work and the world around you. Your “voice” includes how you want your story to be told. If you think your story can be transformed into a chart, one way we can tell your story is by transforming your story into a chart form. We would work with you to put your story into a chart.
- Putting your story in the form of a chart can be useful because that chart shows people the causes that lead to effects that shape your story. People can take those charts and turn them into a software that we call “policy simulation software”. This means that your story can be made part of a computer program that can allow you to show people how their decisions impact your story. This can be a positive or negative impact. You can share this software with others in your community and with people who make decisions that can affect your story: decision makers, policy makers, government, non-profit organizations, and others. You or others from your community can use the chart of your story or also the policy simulation software to advocate for resources and services that

make sense to your community and its realities. Decision makers like politicians or officials at the district or region or national levels can use your voices to know which type of services are important for you and how you want those services to be given to you and what services you want to improve in your community. But it all starts from your voice, your realities, and your lived experiences.

- INTRODUCING NARRATIVE COLLECTION / VIGNETTE FORMATION: The first step that we will do is to hear from you about your life. Tell us about you, your community, your relationships, your journeys, and the experience of poverty in that journey by responding to the following prompt: *Keeping in mind the diversity of people and experiences in your community, write up to 5 vignettes. Each vignette should describe the lived experience of 'poverty' of a fictional individual that can be a composite or anonymous case representing a sub-segment of the community.*

Narrative / Vignette Enrichment Script:

Context: After participants finished developing their initial set of vignettes

Purpose: Enrich vignettes to clarify identify factors that shape the individual's and community's realities. Includes both an identification of barriers as well as an asset framing approach.

Time required during session: 2-3 hours

Materials needed:

- Voice recorder
- Laptop
- Notebook or blank paper
- Pens or pencils

Inputs:

- Series of vignettes or narratives

Outputs:

- An enriched series of vignettes or narratives

Roles: Facilitator to introduce session and process; Community member or key informants to use lived experience to add to and provide feedback on vignettes

Steps: Each of the following can be adapted to match local vocabulary and language of the community. The facilitator introduces the purpose of the activity and the process of enriching vignettes. The following may be one way to do an introduction:

- INTRODUCING NARRATIVE / VIGNETTE ENRICHMENT:
    - The first step that we will do is to listen to and discuss the vignettes that you created. You can add to the vignettes by responding to the following prompt: *Within the context of each vignette, what are positive aspects of the community (such as aspirations, identities, strengths, histories, resources, etc.) that uniquely define? How do those factors interact with an experience of poverty?*
    - To assist participants in marshalling thoughts, some of the following open-ended prompts can be used:

- PROMPTS THAT CENTER THE PERSON & HELPS CREATE AN ASSET-FRAMING NARRATIVE (prompts are disaggregated, but a narrative integrates them and captures the influence of the others on whichever topic is being prompt):
    - What were your dreams and aspirations when you were young? How have that changed over time? What shaped that change? What are some challenges to achieving your dreams and aspirations? What are things in your life that help you to overcome barriers to your dreams and aspirations?
    - What kind of play, schooling, or work have you been involved in? What did you want to be involved in? *If there is a difference between what participant wanted and what they were involved in:* what led to that difference between what you wanted and what you got involved in? how have you adapted to get do what you want to do? What are some things in your life or community that help you to participate in play, schooling, work and other parts of life in the way you want?
    - The table below acts as a further guide for prompts to explore assets in depth, their meaning to the person, their dynamic nature, and their inter-relationships.

**DOMAIN**	**PROMPTS**
**External Resources:** Income, employment, access to credit; housing/shelter; health insurance; infrastructure: internet access & adoption; transportation, food systems, etc; determinants of access to and utilization of resources (e.g. policies, norms, physical barriers)	EXPLORING CONCEPT OF EXTERNAL RESOURCES + CHANGE OVER TIME:<br>• What are external resources that are important to you?<br>• What are conditions that you may have experienced that helped or been barriers in you getting external resources?<br>• How have your external resources changed over time?<br><br>EXPLORING FUNCTION OF EXTERNAL HEALTH & WELL-BEING + CHANGE OVER TIME:<br>• How are your day-to-day / daily activities shaped by the external resources you have? How has that changed over time?<br>• How have your external resources shaped you into who you are now? How has that changed over time?<br>• What barriers do your external resources help you overcome? How?<br>• What challenges do lacking some external resources lead to? How?<br><br>INTERCONNECTIONS WITH OTHER DOMAINS OF OPPORTUNITY TO FORM A RICHER NARRATIVE:<br>• How does the state of your external resources lead to: Internal resources? Community? Internal health & well-being? External health & well-being?<br>• How does {internal resources, community, external health, internal health} help you improve or challenge the state of your external ternal resources?<br>• Describe how the relationship between your external resources and {internal resources, community, external health, internal health} started or changed over time?

**Internal Resources:** Education, skills, talents, artisanship, decision making abilities, etc.; determinants of access to and development of internal resources (e.g. policies, norms, traditional lifeways); innovativeness	EXPLORING CONCEPT OF INTERNAL RESOURCES + CHANGE OVER TIME:<br>• What are internal resources that are important to you?<br>• What are conditions that you may have experienced that helped or been barriers in you getting internal resources?<br>• How have your internal resources changed over time?<br><br>EXPLORING FUNCTION OF INTERNAL RESOURCES + CHANGE OVER TIME:<br>• How are your day-to-day / daily activities shaped by the internal resources you have? How has that changed over time?<br>• How have your internal resources shaped you into who you are now? How has that changed over time?<br>• What barriers do your internal resources help you overcome? How?<br>• What challenges do lacking some internal resources lead to? How?<br><br>INTERCONNECTIONS WITH OTHER DOMAINS OF OPPORTUNITY TO FORM A RICHER NARRATIVE:<br>• How does the state of your internal resources lead to: External resources? Community? Internal health & well-being? External health & well-being?<br>• How does {external resources, community, external health, internal health} help you improve or challenge the state of your internal resources?<br>• Describe how the relationship between your internal resources and {external resources, community, external health, internal health} started or changed over time?
**External / health & well-being; Quantity & quality of life:** life expectancy, chronic disease, recovery from / after illness (cancer, addiction, etc), quantity and quality of life, safety & security, etc; historical & present-day trauma	EXPLORING CONCEPT OF EXTERNAL HEALTH & WELL-BEING + CHANGE OVER TIME:<br>• What does "health" mean to you?<br>• What are conditions that you may have experienced that hurt or help your physical health?<br>• How has your physical health changed over time?<br><br>EXPLORING FUNCTION OF EXTERNAL HEALTH & WELL-BEING + CHANGE OVER TIME:<br>• How are your day-to-day / daily activities shaped by your health? How has that changed over time?<br>• How does your physical health and well-being change over the course of the year?<br>• How has your physical health and well-being shaped you into who you are now? How has that changed over time?<br>• What barriers does your physical health and well-being help you overcome? How?<br>• What challenges does your physical health and well-being lead to? How?<br><br>INTERCONNECTIONS WITH OTHER DOMAINS OF OPPORTUNITY TO FORM A RICHER NARRATIVE:

	• How does the state of your external health and well-being lead to: External resources? Internal resources? Community? Internal health & well-being?<br>• How does {external resources, internal resources, community, internal health} help you improve or challenge the state of your external health and well-being?<br>• Describe how the relationship between your eternal health and well-being and {external resources, internal resources, community, internal health} started or changed over time?
**Internal Health & Well Being:** Self-Actualization / Validation / Meaning to Life (happiness, making a difference in the world, reason for existence, sense of meaning and validation in relation to others, sense of meaning and validation in relation to self, feeling of relevance, mental health, addiction / diseases of despair, etc); agency, identity, empowerment; healing & resilience	EXPLORING CONCEPT OF INTERNAL HEALTH & WELL-BEING (i.e. mental health, spiritual health) + CHANGE OVER TIME:<br>• What does "health of the mind or heart or spirit" mean to you?<br>• What are conditions that you may have experienced that hurt or helps the health of your "mind or heart or spirit"?<br>• How has the health of your "mind or heart or spirit" changed over time?<br><br>EXPLORING FUNCTION OF INTERNAL HEALTH & WELL-BEING (i.e. mental health, spiritual health) + CHANGE OVER TIME:<br>• How are your day-to-day / daily activities shaped by your internal state of being? How has that changed over time?<br>• How does your internal health and well-being change over the course of the year?<br>• How have your internal health and well-being shaped you into who you are now? How has that changed over time?<br>• What barriers does your internal health and well-being help you overcome? How?<br>• What challenges does your internal health and well-being lead to? How?<br><br>INTERCONNECTIONS WITH OTHER DOMAINS OF OPPORTUNITY TO FORM A RICHER NARRATIVE:<br>• How does the state of your internal health and well-being lead to: External resources? Internal Resources? Community? External health & well-being?<br>• How does {external resources, internal resources, community, external health} help you improve or challenge the state of your internal health and well-being?<br>• Describe how the relationship between internal your internal health and well-being and {external resources, internal resources, community, external health} started or changed over time?
**Community & Relationships:** Social Capital / Social Resources; Network / Relationships (parents, siblings, family, friends,	EXPLORING CONCEPT OF COMMUNITY + CHANGE OVER TIME:<br>• What does "community" mean to you?<br>• What are some ways to describe relationships?<br>• Who or what gets the name for you: father, mother, brother, sister, son, daughter, uncle, aunt, friend, family, teacher, mentor, student, mentee?

society/other people, sense of community, connection to people who can link them to internal or external resources, other relationships; connectedness that can lead to opportunity); culture; norms that guide / dictate behavior, status, access to opportunities; traditional trade relationships; traditional knowledge & wisdom transfer; healing & resilience; support systems (intra- and inter-family; intra- and inter-community; ally-ships); leadership & influencers; social innovators and social innovation; inter-generational synergies (communities across time)	• Who are the different types of people that form your community?<br>• What are communities that you are a part of? How has that changed over time?<br>• Which communities see you as a part of them? How has that changed over time?<br><br>EXPLORING FUNCTION OF COMMUNITY + CHANGE OVER TIME:<br>• How are your day-to-day / daily activities shaped by community and relationships? How has that changed over time?<br>• How does who you interact with change over the course of the year?<br>• How have the various relationships around you shaped you into who you are now? How has that changed over time?<br>• What barriers does community help you overcome? How?<br>• How does community provide support?<br><br>INTERCONNECTIONS WITH OTHER DOMAINS OF OPPORTUNITY TO FORM A RICHER NARRATIVE:<br>• How does community support lead to: External resources? Internal resources? External health & well-being? Internal health & well-being?<br>• How does {external resources, internal resources, external health, internal health} help you getting access to and participating in community?<br>• Describe how the relationship between community and {external resources, internal resources, external health, internal health} started or changed over time?

- The facilitator goes through each sentence or discrete thought in the narrative or vignette and uses one or more of the following prompts to explore the system shaping the lived experience of the person and their community's realities:
  - What led to that particular situation being described?
  - Reflection on the cause of the particular situation being described.
  - Reflect on the impact of the particular situation being described.
  - Reflect on questions on the extent to which that particular situation causes or is caused by each of the elements in turn from the Williams-Mohammed Framework
  - Explore each of the following topics from the Williams-Mohammed Marginalization and Health Framework (see body of article for illustration) as leading to or being the result of connections made in the segment being explored of questions on the extent to which that particular situation causes or is caused by each of the elements in turn.
    - Impact of social, economic and other forces on self (e.g. internalized marginalization and discrimination and sense of self, aspirations, expectations, relationship with: policy, systems, etc.)

- Discrimination, inclusion, identity (e.g. othering, ideology of inferiority, institutional, cultural)
- Individual marginalization or discrimination
- Physical marginalization or separation from resources / opportunities / advantages or advantaged communities
- Stereotypes, Implicit and explicit bias, stigma
- Socio-politico-economic forces on community
- Social, economic, and other forces that shape the community (perceived or actual) (e.g. history and historical events, place and environment, political, legal, economic, religious, cultural)
- Impact of social, economic and other forces on community structure (e.g. family structure, segregation, displacement, disenfranchisement)
- Impact of socio-politico-economic forces on individual resources
- Individual and collective resources, social resources
- Impact of socio-politico-economic forces on community resources
- Impact of social, economic and other forces on community resources (e.g. access to resources, transportation, quantity and quality of health, access to healthcare, income, employment, underemployment, wealth, incarceration, civic engagement, other determinants of opportunity)

Narrative / Vignette Analysis Script:

- **Context:** After narrative / vignette is recorded and enriched, narrative analysis can begin
- **Purpose:** An in-depth exploration of enriched vignette and narrative.
- **Time required during session:** 2-3 hours
- **Materials needed:**
  - Voice recorder
  - Laptop
  - Notebook or blank paper
  - Pens or pencils
- **Inputs:**
  - Enriched community narrative or vignettes
- **Outputs:**
  - Enriched narrative or vignette
- **Roles:** Facilitator to introduce session and process; Community member or key informants to voice lived experience
- **Steps:**
  - INITIAL CODING / BREAKING UP THE DATA INTO DISCRETE SEGMENTS: What are some discrete segments that can be identified in this narrative? How can we break apart the vignette into parts that each communicate a distinct idea?
  - INTERMEDIATE CODING: Thinking about the current segment, what is the general idea being conveyed here? How might we summarize the idea being conveyed with a short description or label? How can we look across some of these labels to re-label them or to collapse them together into a single label (i.e. constant comparative analysis technique)? Are there

abstractions that can represent categories of labels? What are relationships between categories? What are cause-effect relationships that we can diagram to connect categories? What other concepts connect to the one we are examining? How do other segments relate to this one? What leads to this? What does this lead to?
    - ADVANCED CODING: In thinking across vignettes, are there common concepts and relationships that can be thought of a certain type of 'category' of experiences? What is the shape of the conceptual model as we synthesize together the relationships we note within each vignette into an 'architecture of systems map' of relationships that represent the spectrum of vignettes?
    - The above is an iterative approach between analyzing content and letting output of the analysis guide generation of additional content.

Architecture of Systems Conceptual Model Building Script:

- **Context:** After conducting narrative analysis, the facilitator will have summarized and visualized the relationships surfaced in the narrative analysis effort. This visualization represents the architecture of systems conceptual model. Now the architecture of systems conceptual model is examined with the participant to ensure that it accurately represents their narrative thoughts.
- **Purpose:** A validation of the summarization and visualization of systems and relationships as mentioned by participant in their narrative or vignette.
- **Time required during session:** 2-3 hours
- **Materials needed:**
    - Voice recorder
    - Laptop
    - Notebook or blank paper
    - Pens or pencils
- **Inputs:**
    - Initial architectures of systems conceptual model (visualization)
- **Outputs:**
    - Validated architecture of systems conceptual model
- **Roles:** Facilitator to introduce session and process; Community member or key informants to voice lived experience
- **Steps:** The facilitator presents the architecture of systems conceptual model and explains that it is a visualization of relationships identified in the narratives from previous sessions. The facilitator goes through each factor and relationship and asks the participant if the way it is represented accurately reflects their experience or how else the factors or relationships can be modified.

Architecture of Systems Conceptual Model Augmenting Script:

- **Context:** After validating the architecture of systems conceptual model with the community to ensure that it accurately captures the relationships the participant had intended to signify in their narrative or vignette.
- **Purpose:** Augmenting the architecture of systems conceptual model with an evidence base. This step additionally serves to stitch together disparate pieces of existing research and literature about the community into a coherent narrative rooted in the community's lived experience.
- **Time required during session:** 2-3 hours

- **Materials needed:**
    - Voice recorder
    - Laptop
    - Notebook or blank paper
    - Pens or pencils
- **Inputs:**
    - Validated architecture of systems conceptual model
- **Outputs:**
    - Augmented architecture of systems conceptual model
- **Roles:** Facilitator to introduce session and process; Community member or key informants to voice lived experience
- **Steps:** The facilitator presents the validated architecture of systems conceptual model and explains that some of the relationships within the model may have been studied by others in their community. This activity presents an opportunity to use that knowledge base to add or improve or add citations to the conceptual model. The participant is then guided to do a literature review (e.g. literature search, "snowballing" additional studies from initial citations). The participant then annotates the conceptual model with the citation and a note including any information related to quantifying relationships. In the process of the literature review the participant may modify or add to the conceptual model.

Architecture of Systems Conceptual Model Annotation for Agent-Based Model Building Script:

- **Context:** After augmenting the architecture of systems conceptual model with the a literature review, parts of the model can be annotated for use as building blocks of an agent-based model.
- **Purpose:** Generating building blocks for an agent-based model.
- **Time required during session:** 2-3 hours
- **Materials needed:**
    - Voice recorder
    - Laptop
    - Notebook or blank paper
    - Pens or pencils
- **Inputs:**
    - Augmented architecture of systems conceptual model
- **Outputs:**
    - Agent-based model building blocks
- **Roles:** Facilitator to introduce session and process; Community member or key informants to voice lived experience
- **Steps:** The facilitator presents the augmented architecture of systems conceptual model and explains that the model can be turned into a simulation but requires a few pieces of information to be attached to each of the factors. For each factor the facilitator would elicit the following information from the community member to the extent possible (https://www.ncbi.nlm.nih.gov/books/NBK305917/):
        - **PROPERTIES:** characteristics of individual agents (such as sex, age, disease state, impairment, disability, wealth, debt, employment status)

- **ACTIONS:** in relation to the factor under consideration, what are the repertoire of specific behaviors that agents can perform within the simulation? (e.g. using public transport, moving around the environment, eating food, smoking tobacco, communicating information to a neighbor, forming a friendship tie)
- **RULES:**
  - What are the rules that connects each factor with its cause? What are the rules that connect each factor to its effect? How do agents relate to each factor: what choices can they make? What properties of each agent is updated? How do the agents interact with the environment or with each other in relation to the factor being examined?
  - What is the current or past value of properties (agent's own properties, those of others, or those of the environment) that forms the input and that needs to be updated for each unit time?
  - Over time, how does factor under consideration relate to agent properties that are important to be updated (level of function, independence, quality of life, health care use, etc)?
- **TIME:** what happens with each pass by the computer through the set of instructions that embody the simulation?
- **ENVIRONMENT:** the context for agents and their interactions in the model: geography, agent types with their own properties, actions and rules, and how they may change with each change in unit time. These would be considered in the context of the particular factor being examined: what type of environment is necessary to represent the particular factor and its relationships being examined?
