# Supplementary material for: Centering and collaborating with community knowledge systems: piloting a novel participatory modeling approach
Source: Int J Equity Health. 2023 Mar 13;22:45. doi: 10.1186/s12939-023-01839-0 (PMC10010640; doi:10.1186/s12939-023-01839-0)
Supplement: Supplementary file 2 — Additional file 2. Output of participatory modeling scripts. [file 12939_2023_1839_MOESM2_ESM.pdf]

SUPPLEMENTAL MATERIAL 2:

OUTPUTS OF PARTICIPATORY MODELING SCRIPTS

Output of Narrative / Vignette Eliciting & Development Script:

The Community Elder

This person is a 65-year-old male who was born and raised in Baltimore. His mother and his grandmother, who both raised him, worked as a domestic aid. His father divorced his mother at an early age. He has a younger brother and sister. While his mother did not have a formal education, she was determined that her children would obtain an education. As such, school was always a priority in his home. This pastor did well in school. He finished high school, received a scholarship to a local college and then received a scholarship to attend a local graduate school where he earned an MBA. His younger siblings did not do so well. His younger brother dropped out of school and got involved in the local illicit drug culture and died at the age of 17. His sister finished high school but became pregnant shortly thereafter. She began working as a waitress at a local restaurant to support herself and new baby.

After graduate school he felt compelled to "help his community" and chose to give up lucrative job offers in the big city to stay home and "fix" the system. He was soon elected to the city council and learned that "politics" often got in the way of progress. He married his high school sweetheart who became a nurse. They had 2 children. Despite his best efforts, his focus on the community, took him away from home often and contributed to his son's rejection of God and the church life. He became part of a gang and was incarcerated for burglary as part of a gang initiation rite.

Given his personal experience and community experiences he decided the best way to help his community was to start a community engaged church which he founded at the age of 35, even though he has no formal seminary or religious training. To try to address the needs of his congregants, he quickly developed partnerships local CBO's. These include Meals on Wheels for meal Services, Immigrant Resettlement Center for a variety of personal housing and workforce development services. Goodwill for clothing, housewares & furniture needs. House of Ruth for domestic violence services, Fraternal Order of Ex-Offenders for ex offender community reentry services, Manpower for employment & workforce training services, Alcoholics Anonymous/ Narcotics Anonymous for Substances abuse services, Women's, Infants and Children (WIC) for nutritional and newborn assistance. The pastor also offered counseling services free to most congregants who wanted it.

His parishioners are largely from the inner city. Most do not have a college degree and therefore work entry level minimum wage jobs. Several have prior incarceration histories mostly for substance abuse and petty theft. He is considered the community spiritual leader.

Over the years he has wanted to expand his services to more community members but to do that he would need to construct additions to the building and make much needed repairs to his church. However, he was unable to secure bank financing and was told that banks do not make loans to his community. Additionally, because of the drug and crime in the neighborhood, city garbage services and even policing are largely nonexistent. As a result, a significant rodent infestation problem has arisen in the alley behind the church. The Pastor is doing his best to eliminate the problem with over-the-counter pest control solutions, but these are increasingly ineffective. The Pastor has tried to get the city to "clean up its act" but has not been successful to date. As a result, the membership is dropping, and the

pastor is having difficulty recruiting a young new pastor to join the church as the new pastor after he retires.

The Community Activist

This is a 52-year-old African American Female. She completed high school and 2 years of college before dropping out to spend full time working for a nonprofit that works to reduce childhood poverty. For the last 15 years she has run a small Community Based Organization (CBO) in the heart of the inner city, that provides after school activities and mentorship for inner city children. She has been diagnosed with bipolar disorder and has been certified as totally disabled. However, she no longer takes any medication because she does not think she needs it. This has resulted in her being hospitalized several times over the last few years. During these times, all activities at her CBO ceased.

She can support the CBO through grant funding from local foundations. This funding is small and episodic. She cannot afford to hire any staff. However, through a partnership with the local university, she has been able to have several students studying childhood education and/ social work to volunteer as interns to work with her on programming for the children and grant writing. The local university gave her a one-time small grant that enabled her to start a youth developed community newspaper that ran for a few years. However, the paper never generated significant revenue and eventually had to be stopped. In the past she has partnered with the local university on a few large research grants. However, these resulted in relatively small personal honoraria for her time, with no significant benefit for the CBO or the community. Over the last 15 years many community children have spent time in one or more of her programs. Some find her building to be a safe space they can come to get away from other troubles. Several of the children who have gone through her program have gone on to complete high school and college. Some come back and donate funds and/or time to the CBO. they insist that her programs helped them learn that the way of the "streets" was not the only way. This helped them to stay in school and complete their education. As a result, she has received several personal and organizational awards and certificates of recognition. She proudly displays each one on the walls of her "kids shelter" as she calls it.

The Blue Middle

Joe is a 48-year-old African American married male who earned a High School diploma and a Vocational-Technical certificate in Halting, Air-conditioning and Ventilation systems (HVAC). While he joined the multistate HVAC repair company where he completed his HVAC internship and has been with the company for the last 20 years, his wife is a full-time home maker who also takes care of her elderly mother who lives with them. He also financially supports his own elderly parents who live in another state. They are both retired and live on fixed incomes. He is a very dependable worker who has not missed a day of work since he started. He has worked his way up to become a mid-level manager. Given his salary and work history he was able to recently purchase a small single-family house in a working-class neighborhood. Because he manages is finances well, he can take his family on modest vacations every 2-3 years. They own one car and maintain a savings account of approximately $3500. He has no investments but does have an IRA that is worth about $50,000. He is working to pay off his new home mortgage, auto loan and parent plus educational loans he secured for his two children to attend college and graduate school. However, he is the first to admit that despite their achievements, they are really one paycheck away from disaster. His debt-to-income ratio is high so, his car and home were both financed at a high interest rate. To make ends meet at the end of the month he has had to use his credit

cards to pay some bills and then work to pay those off by working some extra shifts and getting overtime pay at the company. While he has thus far been able to hold things together, he is worried because with the cost of everything going up each year, it is getting harder to stay afloat.

The Student

Jim was a 25-year-old African American second year law student at at the state university law school. Upon finishing law school, he planned to run for statewide public office and work to disrupt the school to prison pipeline that disproportionately impacts the lives and livelihoods of African American males. Jim's older brother recently completed Medical School. Jim's father is a research scientist, and his mother is an elementary school teacher. His parents were both first generation immigrants from West Africa who, in turn helped all their siblings obtain US citizenship. Despite coming from a stable relatively solidly middle-class family Jim, has seen and experienced injustice many times. Jim's father experienced discrimination in graduate school when he was denied top grades that he deserved, simply because he was African American. In Law school, Jim has the highest GPA among his classmates, yet he was not allowed to be the director of the prestigious Law Review Journal most likely because he is also a practicing Muslim. Being the director of the Law Review is usually required to receive job offers from most prominent and lucrative Law Firms upon completion of Law School. It is notable that in the 135-year history of the Law School, African Americans were not admitted to the school until 1970, the school has never had an African American Valedictorian or director of the Law Review. Jim is also living and going to school in a town where there is a lot of African American Activism. Recently Jim has begun attending and supporting some of the marches and demonstrations. The law school however has only a small minority of African American students and although the activism has all been peaceful to date, has indicated that participating in this form of activism could result in being released from the Law School. Jim is very idealistic and does not understand why this suppression of free speech is allowed. He was unceremoniously discharged from law school without the opportunity to appeal. He was also informed that his permanent HR file will include that he was displaying behavior unbecoming of the Law School and a prospective Law Professional. Jim has subsequently applied to 3 other law schools and several law firms that have strong racial discrimination law practices, all to no avail. Jim is currently driving for Uber to make ends meet while he determines his next step.

Output of Narrative / Vignette Analysis Script:

The Community Elder

> This person is a 65-year-old male who was born and raised in Baltimore.
>
> [born & raised in B'more <--- steel industry in 40s, 50s, 60s, brought family to B'more...when steel industry went away, many white people who were able to, moved away (Mt. Washington), AAs who didn't have as many resources, stayed ---> [early on both whites & blacks couldn't move away][people who couldn't live in more affluent part of town, moved to poorer parts and couldn't move away once there...stayed in East and West B'more] <--- block-busting [facilitated one black family moving into a neighborhood, led to White people moving out, real estate investors bought out the homes at a low cost] <--- as steel industry diminished, led to change in economic & social fabric...happened in other places as well (e.g. Birmingham, etc)] <-- basic financial opportunities went away because steel industry went away, led to others who took advantage at the detriment of AAs and profited from racial tensions that led to block busting

(change in economics + racial tension ==> white flight from B'more w/ resources, concentration of poverty, loss of jobs)

His mother and his grandmother, who both raised him, worked as a domestic aide. His father divorced his mother at an early age.

[Grew up in single parent home w/ parents that were not highly educated; mother determined education was the way out of poverty...didn't have lot of exposures or access to opportunity early on: one of the siblings was able to achieve academic success, but younger brother did not...diversity of outcomes of children coming from the same environment and making poor choices can happen in poor as well as wealthy communities...but the consequences of are different: in a poor community a gang will catch the child that is falling through from the family; in a wealthy community there may be other structures that catch them as they fall through and have existential crises in search for meaning --> diversity of outcomes happens in both communities, but in one community it leads to death, loss of hope, and disadvantage, in the other it can lead to temporary setbacks]<-- loss of hope: even if opportunity presents itself then person doesn't take advantage of it because they don't see that opportunity as opportunity for them, don't see themselves as getting to the end or outcome of the opportunity, causes person to question themselves about whether it can actually be done; proud when they see others from community achieving advantage of opportunity, but don't see themselves in that role or outcome of success at the end of opportunity (e..g as a doctor)] ===> CONSERVED MODULE: all communities experience similar susceptibility to individual choices of good or bad choices...but the consequences, off-ramps, remediation, and subsequent opportunities are different, which leads to a much greater impact of poor choice and much less impact of good choices in disadvantaged communities vs advantaged communities

*****[interventions need to take into account both actualizing hope AND opportunity...or else even if opportunity is made available, loss of hope prevents taking effective advantage of it]*****

He has a younger brother and sister. While his mother did not have a formal education, she was determined that her children would obtain an education.

[mother's father worked in steel mill...children raising themselves --> environment has much more of an impact; saw parents divorced, mother didn't work and was not educated, so daughter didn't feel need for education, got pregnant early, led to dropping out of school and not completing her education: generational cycle of poverty]

As such, school was always a priority in his home. This pastor did well in school. He finished high school, received a scholarship to a local college and then received a scholarship to attend a local graduate school where he earned an MBA. His younger siblings did not do so well. His younger brother dropped out of school and got involved in the local illicit drug culture and died at the age of 17. His sister, finished high school but became pregnant shortly thereafter. She began working as a waitress at a local restaurant to support herself and new baby.

[DIVERSITY OF OUTCOMES MORE ACCURATELY REPRESENTS REALITY: older sibling broke through, younger two siblings did not]

After graduate school he felt compelled to "help his community" and chose to give up lucrative job offers in the big city to stay home and "fix" the system.

"SYSTEM" --> confluence factors that are negative influences surrounding his community and create disadvantage and are barriers to advantage.

He was soon elected to the city council and learned that "politics" often got in the way of progress.

He married his high school sweetheart who became a nurse. They had 2 children. Despite his best efforts, his focus on the community, took him away from home often and contributed to his son's rejection of God and the church life. He became part of a gang and was incarcerated for burglary as part of a gang initiation rite. [more advantaged community may also have absent father and resentment, etc...and children would still get involved in drugs, etc, but the consequences would be very different: children in more poor communities may get involved in gangs; in affluent communities there may be sufficient safety nets that catch children when they make mistakes]

Given his personal experience and community experiences he decided the best way to help his community was to start a community engaged church which he founded at the age of 35, despite the fact that he has no formal seminary or religious training. To try to address the needs of his congregants, he quickly developed partnerships local CBO's. These include Meals on Wheels for meal Services, Immigrant Resettlement Center for a variety of personal housing and workforce development services. Goodwill for clothing, housewares & furniture needs. House of Ruth for domestic violence services, Fraternal Order of Ex-Offenders for ex offender community reentry services, Manpower for employment & workforce training services, Alcoholics Anonymous/ Narcotics Anonymous for Substances abuse services, Women's, Infants and Children (WIC) for nutritional and newborn assistance. The pastor also offered counseling services free to most congregants who wanted it.

***ENABLING INSTITUTION: [Historically, church was only place where AA can gather together, communicate freely, and receive support...church was a place where you could hear more than about God...spirituals that were sung communicated not only spirituality but code on the pathways of the underground railroad, about freedom, etc] --> church becomes a source of help and strength...this included enabling access to determinants of opportunity (aspects of the ecosystems of opportunity metamodel): CBOs, meals on wheels for services, immigrant resettlement center, goodwill for clothing, houseware & furniture, domestic violence services, etc.

His parishioners are largely from the inner city. Most do not have a college degree and therefore work entry level minimum wage jobs. Several have prior incarceration histories mostly for substance abuse and petty theft. He is considered the community spiritual leader.

*** over-policing --> elevated arrests for crimes, convictions, incarcerations --> predominant number of AA males have a record in some communities for non-violent crimes

Over the years he has wanted to expand his services to more community members but to do that he would need to construct additions to the building and make much needed repairs to his

church. However, he was unable to secure bank financing and was told that banks do not make loans to his community.

Additionally, because of the drug and crime in the neighborhood, city garbage services and even policing are largely nonexistent. As a result, a significant rodent infestation problem has arisen in the alley behind the church. The Pastor is doing his best to eliminate the problem with over-the-counter pest control solutions, but these are increasingly ineffective. The Pastor has tried to get the city to "clean up its act" but has not been successful to date. As a result, the membership is dropping, and the pastor is having difficulty recruiting a young new pastor to join the church as the new pastor after he retires.

The pastor believes that the local financial system including the banks are working against people from his community. This is evidenced by his inability to obtain a construction or rehabilitation loan to enhance the church, despite demonstrating the ability to make adequate monthly payments. In addition, extremely high interest (2500%) personal loans are easily given to residents of the community, effectively draining the community via interest payments and a variety of additional fees.

Because the church is located in an inner city "redlined" area where local banks charged African Americans and Hispanics higher rates and fees on mortgages even when they qualify for better deals primarily by steering minority borrowers into subprime mortgages even though they qualify for cheaper loans. The Pastor believes this same kind of thinking has precluded them from being able to secure a loan to rehab and expand the church. Given the pastor's situation, he relies heavily on his ability to provide counseling services to nonmembers of his congregation. While he does charge these clients, because of their limited ability to pay, his fees are generally considered low. He tries to compensate by working longer hours and seeing as many clients as possible. He also works to submit grant applications to local organizations providing small grants to local CBO's that provide critical services.

Given the pastor's situation, he relies heavily on his ability to provide counseling services to nonmembers of his congregation. While he does charge these clients, because of their limited ability to pay, his fees are generally considered low. He tries to compensate by working longer hours and seeing as many clients as possible. He also works to submit grant applications to local organizations providing small grants to local CBO's that provide critical services.

The hospital where the pastor's wife work has been a good supporter of the church and community. While they cannot provide health insurance for those who do not have it. They provide free health screening days at the church twice a year. At these events the members can get a variety of health screening tests performed free of charge. If problems are detected, efforts are made to identify resources to pay for needed care. While helpful in isolated cases, this piecemeal approach to financing of care does little to help the greater community address its health problems which are inextricably tied to their socioeconomic position and household incomes.

The Pastor is a diabetic with hypertension. Because his church does not offer insurance for its employees, he has obtained federally sponsored health insurance. Because he has had to draw from his personal finances to support the church, he has no savings and at times has been

unable to obtain his medications. As a result, both his blood pressure and diabetes are under poor control resulting in hospitalizations at least 2-3 times a year. Despite the government sponsored insurance, these hospitalizations have resulted in large bills which he is responsible to pay. He is unable to pay these debts. This has contributed significantly to his poor personal credit score. This in turn has affected everything from the interest rate he was given for his car, the interest rate on his credit cards the home he was able to buy and even his ability to cosign for loans to help his children attend and finish college.

Given the pastor's personal mission to build the church and help the community and the resulting challenges that he has had to face over the last 30 years, despite holding a respected position in the community, he often suffers from feelings of inadequacy and failure. At times he has sought counseling for "depression" although he refuses any medication. It has also contributed to marital problems that he and his wife continue to work through without let\ting the congregants know. This also contributes to feelings of inadequacy and failure. He is tortured by questions such as "Why can't the respected spiritual leader of the community, actually help improve the community or even keep a happy marriage".

The pastor has expended all his personal savings and has no retirement account. Therefore, he will need to work as long as possible. His wife retired from a nursing career and has a small pension coming when she turns 65 in a few years. Worry about his retirement is another source of constant stress.

Many CBOs with which the Pastor has chosen to collaborate in order to bring needed services to his community remain small and underfunded and can therefore only offer relatively limited scopes of services. Also, because many needed goods and services are not accessible by many in the community, a vibrant "underground" economy thrives. It largely operates person to person, word of mouth, on the street or in private homes. There are no formal stores or shops, no traditional advertising, and no interaction with the existing state of federal financing and retail structures. Here residents create and sell products, provide a wide variety of services, paid for through a combination of cash, reciprocal services or other valuable goods. The Pastor has often used this alternate economy, to get things done around the church.

The Community Activist

She has worked hard to bring resources to children in the inner city, however these resources are limited. Because she has a mental health condition, her program is not able to qualify for any of the federal programs (Head Start, Healthy Start etc.) designed to support children. Also, the local university always seems interested in doing research about the community, but this never results in any significant resources coming to the community. Local philanthropies have been helpful, but the resources have been small and inconsistent. All these factors conspired to prevent substantial growth of the CBO.

Because this program is in the inner city many of the children, she has worked with live in poor housing conditions and suffer from inadequate nutrition. Because of her limited education and medical condition, she is not able to work another job that would bring in more income. Her home is home was built in the 1950's and therefore has a lot of lead-based paint and asbestos in the home. Because she is unable to buy a home and must rent, she has limited housing

opportunities that fit into her budget. she worries that the children coming to her building may be affected by the lead-based paint or asbestos. She has spoken to her landlord about this many times, but the landlord refuses to do anything. She does not own a car and as a result is defendant of public transportation which is very unreliable in her part of the city. As a result, she is limited in the number of groceries she can buy because she has to carry everything with her on the bus after shopping. She does have an internet connection for the children to use, but it is a DSL line. The faster cable and broadband services are too expensive for her to buy a subscription.

She is a very creative person who has no lack of ideas for projects that her after school children can work on. These have included developing a community newspaper, a variety of arts and crafts projects, reading/book club, cooking classes and homework support. This skill has been useful when applying for grants to support the program. Many of her funded applications have been deemed as very innovative, with significant potential impact. She also has an amazing memory. This has enabled her to become somewhat of the neighborhood historian. Her recollection of dates, names and events that have occurred in the community is amazing. Even the local university relied on her when seeking to conduct an in-depth study of the history of the community and its relationship to the university.

Although she has been diagnosed with a mental health condition, she feels she is normal" having up s and downs like everyone else. She also believes that while she does not have all that she wants, she has much of what she needs and has learned to live with what she has. Therefore, she considers her quality of life to be adequate. She is very worried about the children in the community because they are exposed to a significant amount of gun violence, police brutality, drugs, gangs, poor housing and nutrition beginning at very young ages. She believes strongly that these experiences help shape the futures of these children in a negative way. That is why she works so hard to provide as many as possible with positive life experiences that can help motivate them and enhance tier quality of life.

She is concerned because many of the children she works with believe their lives are constrained to only a relatively small number of careers. These largely fall in the minimum wage, entry level categories, construction, music and entertainment industries and sports. While she believes all honest work is valuable and honorable, she works to expand the minds of the children to include nontraditional career paths and life goals. Often though she is only able to talk with them and share books or online content with the children. This is all she can afford. Because these children generally have no one else doing this for them, he understands that she is providing an incredibly valuable and empowering service for these children.

When she exposes the children to the possibilities of carriers in business or owning a business many get excited. The current public educational system which most of the students attend, do not have any courses encouraging them or exposing them to the potential of entrepreneurism or innovation, finance, and banking. Generally, career expectations of these children by their formal teachers are low. That is another reason she works so hard to change this reality in the lives of these children. Typically, the books these children are given in school are old and outdated as are the physical buildings themselves.

This lady often struggles with feelings of inadequacy because she does have a mental health condition and did not complete college herself. She has been told often that she would never "achieve anything significant". She has been led to believe that her business has less value than other more well-funded programs in other neighborhoods. These feelings go away however when she reflects on all the awards and expressions of gratitude and thanks received from former students who have gone on and done well.

Because she is very outspoken on the issues that matter to her and because she has a mental health condition, she has been called "crazy" or "mental". She feels that many opportunities have been withheld from her and her organization because of her condition and lack of completing formal college education, despite her track record of success and evidence of value in the community.

She has often felt the stigma of mental health as well as the stigma of living in the inner city. It is most often expressed in terms of low expectations and doubt of past accomplishments. These in turn lead to pessimism regarding future potential. Collectively these attitudes and beliefs limit her access to capital, educational opportunities, and opportunities to grow and expand her business so that she can serve more children in the community.

Much of her life is spent in the confines of the inner city. She has little ability or opportunity to engage with people or activities in other non-inner-city communities.

Mental health stigma (these people are crazy) as well as stereotypes regarding poverty (poor people are lazy) and inner city/African American (violent, using or selling drugs) are experienced often. As a result, financial organizations often have little trust in her ability to manage finances effectively and be a responsible steward of loans or investments of any significant size.

Retail food establishment and retail zoning laws facilitate large numbers of "cornerstone liquor establishments and small number of sit-down eateries and robust grocery store chains in the inner city. Crime rates and lack of equitable policing, tend to disincentivize businesses from locating offices in the inner city and residents from becoming entrepreneurs. As a result, employment opportunities are minimized, unemployment is elevated, hope is lacking an cynicism is prevalent.

These tend to be mutually reinforcing principles that collectively inhibit change from occurring. This enables these problems to become intransigent and generational.

These factors tend to reinforce poverty, poor health, lack of access to capital and other human resources, motivational activities and experiences that can galvanize change.

Promotes disenfranchisement and cynicism.

The Blue Middle

This is a "successful" middle class working professional who is living in a financially precarious position and under significant financial burden despite being a very dependable, hard worker who has achieved some upward mobility at his job. However, salary increases have not kept pace with increases in cost of living. Significant debt burden is also a chronic concern. His wife's caregiving role precludes her ability to work and adds to her stress from not being able to

"contribute financially". The stress of caregiving is taking toll on her mental health at the same time her husband is working long hours and extra shifts to try to make ends meet.

Given his situation he has no ability to work a second job to increase his income. His wife is also not able to work because of her work as a caregiver. Also because of his significant financial debt, credit options are few and costly. Some states offer limited financial resources to family caregivers; however, they do not live in one of those states. Were this the case, it would be a substantial help.

The home and car are the main family assets. They are used to support the nuclear family and the aging parents.

This working-class neighborhood that he lives in is close knit, with neighbors "looking out" for each other. They support each other by lending a helping hand with hoe repairs, occasionally giving the wife a break to do some shopping by sitting with the elderly parents and offering to help with yard work and household cleaning. This helps keep costs down and 0rovides some periodic stress relief.

Joe is a 1 pack per day smoker who has been smoking since the age of 18. (30 pack years). He also has recently been diagnosed with hypertension which he is attempting to manage via diet and lifestyle changes.

Because he is not a complainer and tends to internalize his feelings, he states that he is "as healthy as the next guy". His only wish is that he had more time to spend with his family.

Joe's only interaction with the financial system has been in the context of buying his home and cars. He also has three credit cards. Because of his debt situation, his interest rates are relatively high, and he has a lower credit score, limiting his access to credit.

Joe has thought about starting his own HVAC company, but his tight current financial system, lack of access to credit and minimal family savings has kept him from attempting to start the business.

Because most of Joe's financial debt is due to student loan debt, he feels it is a little unfair. Without the student loans, his children could not complete their educations and be able to get ahead. But because he was working to take care of his family and help his children get ahead, it now inhibits his ability to grow, expand and support his family and aging parents without spending even more time away from home working.

Although Joe is successful compared to many of his childhood friends, he feels that his professional friends and associates talk down to him because he only has a high school degree and technical training. The financial institutions see his earning potential as moderate and do not consider his work ethic or dependability.

Stereotypes: “Working class families are not very bright and have limited earning potential.”

Given national sociopolitical conditions local community members are very "protective" of their community. This meant that there was real resistance to Joe and his family when they bought

their house and originally moves into the largely Caucasian community. Some members still refuse to accept Joe as a legitimate member of the community.

Community members seem less willing to help Joe and his family, in the same ways they help other families. The HOA has passed new ordinances restricting ethnic public expression, loud noise/music, or large gatherings (parties) without prior approval of the association. Joe feels sure that these were passed because of discriminatory views of his new neighbors.

The Student

In this persona, we find religious systems intersecting with educational systems, social systems, political systems which ultimately impact financial opportunities. Here religious discriminations combined with racial bias collude to preclude educational attainment and thereby significantly limit financial opportunity. The vantage point is from a professional student with significant promise.

As a promising Law student, Jim was already being given access and prepared for future access to credit, housing resources, business opportunities, mentorship all of which led to significant economic opportunity for the individual and his/her family, yet all of things were taken from Jim leaving him and his future family will significantly reduce economic/financial opportunities. In addition, the reputational assessment in the form of the HR personnel file will forever follow him and potentially always negatively affect his income/earning potential.

Given cumulative law school GPA, Jim demonstrated significant academic/educational achievement. talent and potential. He has developed enviable critical thinking skills and analytic ability while also perfecting his writing and written communication.

Jim's parents and brother were all hard workers and high achievers and, as a result, Jim was exposed early to the benefits of delayed gratification of desires, focused effort and determination even in the face of challenges and setbacks. These generational traits were passed on to Jim and he will pass them on to his future children.

As a young man Jim's health has generally been good. However as social discord increased in his state he began struggling with issues of depression. He also felt extremely privileged to be in law school and felt an enormous burden to help correct the many systemic wrongs he witnessed in local and national society. This issue grew in magnitude and severity after his release form law school and his subsequent inability to obtain stable employment. The increasing stress contributed to his developing hypertension. However now, he was without health insurance and unable to afford medications or see a doctor regularly.

Over time Jim began to doubt his cognitive abilities and his employability. This contributed to his mental health and physical problems. He soon became very angry at society and cynical. He no longer viewed society as fair.

Given Jim's experience, he felt precluded from robust interactions with the financial system and relegated to inferior financial opportunities including high interest rate credit card, subprime auto loans and excessive mortgage interest rates, predatory loans, and cash only options. Equity

investments, many educational grants and employment based financial incentives were no longer a part of his reality.

As a result of his experiences, Jim increasingly saw himself as unable to be successful in such a biased and racist society. Before long Jim abandoned his goals as pipe dreams that are not achievable in such a racists society. He began to mistrust all levels of government, because he felt they were put in place to provide the appearance of helping while simply perpetuating the injustices.

Jim explicitly experienced discrimination based on his Muslim faith and his African American race/ethnicity. He also often felt many situations while not overtly racist, were much more subjectively structured to cater to those of European descent. For example, he recalls being out walking in his neighborhood, getting some exercise when he was in law school and being stopped and questioned on more than one occasion as to why he was in the neighborhood. He often was asked to explain himself or justify his positions when others in similar circumstances were not required to do the same.

Jim strongly believes that his dismissal from law school for unspecified "behaviors" unbecoming of a future law professional was very discriminatory. He also believes that his inability to gain admittance to another law school or law related employment demonstrates at least in part the same, discriminatory behavior.

Despite growing up and while attending law school Jim live in solidly middle class, integrated neighborhoods, these neighborhoods were usually greater than 70% white/Caucasian homeowners. Since his release form law school the only place he could afford to live is an even more segregated low income community that is 95% African American.

Jim has in the past been called a terrorist, violent, undependable, and untrustworthy despite never exhibiting any of these characteristics or traits.

Historically, African Americans were not permitted to enter law school. While currently, Jim's law school does admit a small number of African Americans, the faculty contains many professors who worked during the era when no African Americans were admitted. Some still harbor strong personal views of the inferiority of students of African American descent. Jim has also been the subject of racial profiling by police and homeowners in the communities in which he lived.

Prior to law school, Jim had come to live with a baseline level of anxiety about race and religious based discriminatory behavior in society. These behaviors seemed not only to have become normalized in society, but Jim's personal reaction to it had become so normalized to him, that he did not perceive and effect on him physically or psychologically. Later though, as the series of events in law school and thereafter occurred, Jim realized that he had a lot of internalized anger and resentment from his personal and societal experiences of inequitable and unjust treatment in society.

The events that occurred in Jim's life initially positively affected his access to individuals' resources. However, the events that followed largely in law school and thereafter significantly contracted and limited the availability of resources Jim could access.

As a result of the events outlined above, Jim now lives in a low income, segregated community. This community has a high number of abandoned properties with broken windows, is located near an urban industrial zone which is a frequent air polluter. His longer-term financial outlook has been severely constricted and has become a source of considerable emotional stress. His employment prospects are complicated by the fact that he is over educated for many positions and still undereducated for others. As a result, he is forced to work minimum wage jobs while he contemplates his next educational goals. These however are also constrained due to his restricted income. Jim remains depressed without medication because he is unable to afford what was suggested at the emergency room where he was taken, when his friends became worried after he became drunk at a party and started talking about ending his life.

Output of Architecture of Systems Conceptual Model Building Script & Architecture of Systems Conceptual Model Augmenting Script

Supplement 2, Figure 1: Architecture of Systems Map (see Figure 3 in article for higher definition image)

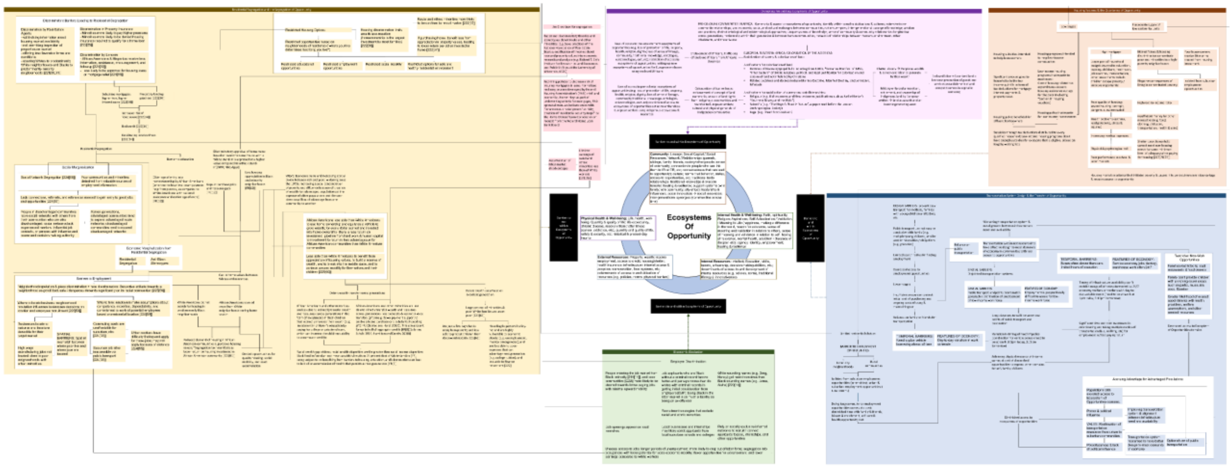

Output of JSON Representation of conceptual model (GoJS 2.2 Output, Northwoods Software)

{ "class": "GraphLinksModel",

"nodeDataArray": [

{"text":"Worsening transportation system & misalignment between infrastructure need and availability","key":-9,"loc":"3138.5 -2791","size":"160 80","fill":"pink","group":-90},

{"text":"Transportation system not resourced to have effective design to meet demands of economy in communities with low access to opportunities","key":-12,"loc":"3133.5 -2651","size":"180 80","fill":"lightblue","group":-17},

{"text":"Reliance on public transportation","key":-3,"loc":"2913.5 -2651","fill":"lightblue","group":-17},

{"text":"Reliance on family or friends for transportation","key":-4,"loc":"3918.5 -2651","fill":"lightblue","group":-90},

{"text":"Limited residential choices","key":-5,"loc":"2268.5 -2651","fill":"lightblue","group":-90},

{"text":"FEATURES OF ECONOMY:\nDay-to-day variation in work schedule","key":-6,"loc":"3993.5 -2531","size":"140 60","fill":"lightgreen","group":-14},

{"text":"TEMPORAL BARRIERS:\nNeed to plan vehicle borrowing ahead of time","key":-7,"loc":"3833.5 -2531","size":"160 60","fill":"beige","group":-14},

{"text":"Variation in timing of need impedes coordination for vehicle access (need to be at work at 1pm today, 3:15pm tomorrow)","key":-8,"loc":"3908.5 -2391","size":"200 80","fill":"lightyellow","group":-90},

{"text":"Significant time and cost investment in coordinating and taking multiple modes of transportation (bus, walking, etc) for employment w/ low paying return","key":-10,"loc":"3508.5 -2201","size":"180 100","fill":"lightyellow","group":-90},

{"text":"Timing of infrastructure availability don't match timings of infrastructure need by 24/7 economy workers or workers with day-to-day variable needs (need to be at work at 1pm today, 3:15pm tomorrow)","key":-11,"loc":"3508.5 -2371","size":"200 120","fill":"lightyellow","group":-90},

{"text":"FEATURES OF ECONOMY:\nService economy jobs; factory, warehouse work often 24/7","key":-13,"loc":"3593.5 -2521","size":"180 60","fill":"lightgreen","group":-16},

{"isGroup":true,"text":"","key":-14,"loc":"3908.5 -2531","dash":[2,4],"group":-90},

{"text":"TEMPORAL BARRIERS:\nBuses often slower than cars; limited hours of operation","key":-15,"loc":"3413.5 -2521","size":"160 60","fill":"beige","group":-16},

{"isGroup":true,"text":"","key":-16,"loc":"3508.5 -2521","dash":[2,4],"group":-90},

{"isGroup":true,"text":"","key":-17,"loc":"3038.5 -2651","group":-90},

{"text":"SPATIAL BARRIERS:\nDisjointed transportation systems","key":-18,"loc":"2818.5 -2521","size":"160 60","fill":"beige","group":-90},

{"text":"DESIGN BARRIERS: prevent easy transport for mothers, families with young children and babies, etc","key":-19,"loc":"2568.5 -2521","size":"180 60","fill":"beige","group":-90},

{"text":"BARRIER TO EMPLOYMENT OPPORTUNITIES","key":-20,"loc":"2268.5 -2521","size":"180 60","fill":"beige","group":-90},

{"text":"Inner-city neighborhoods","key":-21,"loc":"2158.5 -2381","figure":"Terminator","size":"180 40","group":-90},

{"text":"Rural communities","key":-22,"loc":"2348.5 -2381","figure":"Terminator","size":"180 40","group":-90},

{"text":"Isolated from suburban employment opportunities (inner-cities); urban & suburban empl. opportunities (rural comm.)","key":-23,"loc":"2258.5 -2191","size":"260 80","fill":"lightyellow","group":-90},

{"text":"Doing long commute to employment opportunities comes at a cost: diminished time with family & friends; leisure & enrichment; self-care & health; opportunity cost","key":-24,"loc":"2258.5 -2051","fill":"lightyellow","size":"260 80","group":-90},

{"text":"Achieving physical resource of income comes at cost of diminished opportunities to expand other domains for self, family, children","key":-25,"loc":"2888.5 -1911","size":"180 100","color":"brown","group":-90},

{"text":"Families restricted to local restaurants & food deserts","key":-26,"loc":"2993.5 -2081","size":"180 60","color":"brown","group":-78},

{"text":"Public transport endpoints don’t match geographic distribution of employment of low-income work force","key":-27,"loc":"2823.5 -2371","size":"200 80","fill":"lightyellow","group":-29},

{"text":"Public transport endpoints don’t match geographic distribution of employment of low-income work force","key":-28,"loc":"3023.5 -2371","size":"180 60","fill":"lightgreen","group":-29},

{"isGroup":true,"text":"","key":-29,"loc":"2918.5 -2371","dash":[2,4],"group":-90},

{"text":"Long distances to walk between end point of public transport and destination","key":-30,"loc":"2908.5 -2201","fill":"lightyellow","size":"180 60","group":-90},

{"text":"Limited search radius for finding employment","key":-31,"loc":"2568.5 -2201","fill":"lightyellow","group":-90},

{"text":"Public transport use not easy or conducive with family (e.g. multiple young children, stroller use) or necessities / obligations (e.g. groceries)","key":-32,"loc":"2568.5 -2371","fill":"lightyellow","size":"160 120","group":-90},

{"text":"Decreased access to employment opportunities","key":-33,"loc":"2568.5 -2111","size":"180 60","color":"brown","group":-90},

{"text":"Lower wages","key":-34,"loc":"2568.5 -2031","size":"120 40","color":"brown","group":-90},

{"text":"Insufficient resources to meet initial cost of purchasing and ongoing costs of using & maintaining car","key":-35,"loc":"2578.5 -1911","size":"140 100","color":"brown","group":-90},

{"text":"Diminished opportunities to expand external resources: fresh food grocers, outdoor activities, parks, public spaces, etc","key":-36,"loc":"2698.5 -1741","size":"160 100","color":"brown","group":-81},

{"text":"Diminished opportunities to expand community & social network; physical and mental growth & enrichment opportunities (e.g. museums, gyms, parks)","key":-37,"loc":"2878.5 -1741","size":"180 100","color":"brown","group":-81},

{"text":"Diminished opportunities to expand internal resources: leadership initiatives, enrichment activities, innovation and entrepreneurial spaces","key":-38,"loc":"3068.5 -1741","size":"180 100","color":"brown","group":-81},

{"text":"Diminished opportunities to expand mental and internal well-being through enrichment opportunities, museums, gyms, parks, etc","key":-39,"loc":"3258.5 -1741","size":"180 100","color":"brown","group":-81},

{"text":"Diminished access to opportunities for achieving physical health and well-being: waiting for transport in unsafe neighborhood, missed medical appts.","key":-40,"loc":"3448.5 -1741","size":"180 100","color":"brown","group":-81},

{"isGroup":true,"text":"","key":-41,"loc":"3078.5 -1749.66328125","dash":[2,4],"fill":"pink","color":"purple","group":-90},

{"text":"Powerlessness & lack of political influence","key":-42,"loc":"4033.5 -1730","size":"180 40","color":"brown","group":-172},

{"text":"VALVE: Reallocation of transportation resources from urban to suburban communities","key":-43,"loc":"4103.5 -1930","size":"180 80","fill":"white","color":"purple","group":-172},

{"text":"Power & political influence","key":-44,"loc":"4103.5 -2140","size":"180 40","fill":"white","color":"purple","group":-172},

{"text":"Populations with elevated access to Ecosystem of Opportunities domains.","key":-45,"loc":"4103.5 -2240","color":"purple","group":-172,"size":"140 60"},

{"text":"Improving transportation system & alignment between infrastructure need and availability","key":-46,"loc":"4293.5 -2060","color":"black","fill":"lightgreen","group":-172,"size":"140 80"},

{"text":"Transportation system resourced to have better design to meet demands of economy","key":-47,"loc":"4243.5 -1820","size":"160 60","group":-49},

{"text":"Optional use of public transportation","key":-48,"loc":"4423.5 -1820","size":"160 60","group":-49},

{"isGroup":true,"text":"","key":-49,"loc":"4333.5 -1820","group":-172},

{"text":"Low wages","key":-51,"loc":"972 -3910.584","size":"200 40","fill":"lightyellow","group":-53},

{"text":"Inadequate supply of low-cost rental units","key":-52,"loc":"1202 -3910.584","size":"200 40","fill":"lightyellow","group":-53},

{"isGroup":true,"text":"","key":-53,"loc":"1087 -3910.584","group":-89},

{"text":"High mortgage","key":-56,"loc":"887 -3745.584","size":"200 40","fill":"lightyellow","group":-68},

{"text":"Poor quality of housing: severe housing: decrepit, dangerous, overcrowded","key":-57,"loc":"1107 -3735.584","size":"200 60","fill":"lightyellow","group":-68},

{"text":"Poor house owners receive little or no reward from housing investment","key":-58,"loc":"1567 -3745.584","size":"200 60","fill":"lightyellow","group":-68},

{"text":"Market forces & housing discrimination concentrate poor and minorities into high-poverty neighborhoods","key":-59,"loc":"1347 -3745.584","size":"200 60","fill":"lightyellow","group":-68},

{"text":"Lower part of household budget devoted to education, training, child care, healthcare, job searches, transportation, other investments to help children escape poverty / improve opportunity","key":-61,"loc":"887 -3615.584","size":"200 120","fill":"lightyellow","group":-68},

{"text":"Health problems (asthma, lead poisoning, stress & injuries)","key":-62,"loc":"1117 -3645.584","size":"200 60","fill":"lightyellow","group":-68},

{"text":"Increasing medical expenses","key":-63,"loc":"1117 -3565.584","size":"200 40","fill":"lightyellow","group":-68},

{"text":"Physical & psychological toll","key":-64,"loc":"1117 -3495.584","size":"200 40","fill":"lightyellow","group":-68},

{"text":"Poor performance in school & labor markets","key":-65,"loc":"1117 -3425.584","size":"200 40","fill":"lightyellow","group":-68},

{"text":"Isolated from suburban employment opportunities","key":-66,"loc":"1567 -3645.584","size":"200 60","fill":"lightyellow","group":-68},

{"text":"Negative consequences of living in concentrated poverty","key":-67,"loc":"1337 -3660","size":"200 60","fill":"lightyellow","group":-68},

{"text":"Housing market as a factor that initiates poverty & causes it to persist; increases disadvantage & reduces access to opportunity ","key":-69,"loc":"1211 -3251.7519999999995","size":"200 80","fill":"lightyellow","group":-89},

{"isGroup":true,"text":"Lack of Adequate & Affordable Housing: Rental Affordability Crisis","key":-68,"loc":"1227 -3572.792","group":-89},

{"text":"Housing subsidies intended to help home owners","key":-70,"loc":"317 -3740","size":"200 40","fill":"white","color":"purple","group":-89},

{"text":"Significant amount goes to households in the top income quintile in form of tax deductions for mortgage interest payments & property taxes","key":-71,"loc":"317 -3610","size":"200 100","fill":"white","color":"purple","group":-89},

{"text":"Housing policy beneficial for affluent home owners","key":-72,"loc":"317 -3450","size":"200 100","fill":"white","color":"purple","group":-89},

{"text":"Low-income housing programs inadequate to meet need:\nCost of housing related tax expenditures exceeds housing assistance outlays for the poor (including \"Section 8\" housing vouchers)","key":-74,"loc":"561 -3611.752","size":"200 100","fill":"white","color":"purple","group":-89},

{"text":"Subsidies through tax deductions distributed to every qualified household; Low-income housing programs don't have enough subsidies for everyone that is eligible; placed on lengthy waiting lists ","key":-75,"loc":"437 -3280","size":"200 120","fill":"white","color":"purple","group":-89},

{"text":"Housing policy inadequate for low-income home owners","key":-73,"loc":"561 -3451.752","size":"200 100","fill":"white","color":"purple","group":-89},

{"text":"Housing programs intended to help low-income communities","key":-76,"loc":"551 -3741.752","size":"200 40","fill":"white","color":"purple","group":-89},

{"text":"Decreased access to Ecosystem of Opportunities domains.","key":-77,"loc":"3128.5 -1971","size":"180 60","color":"brown","group":-90},

{"text":"Family can't provide children with enriching experiences such as parks, museums, zoos, libraries","key":-79,"loc":"3193.5 -2081","size":"180 60","color":"brown","group":-78},

{"text":"Greater likelihood of missed appointments with health providers, welfare caseworkers, and other needed resources","key":-80,"loc":"3383.5 -2081","size":"180 60","color":"brown","group":-78},

{"isGroup":true,"text":"Restricted Non-Work Opportunities","key":-78,"loc":"3188.5 -2081","group":-90},

{"isGroup":true,"text":"Dimensions of Opportunity","key":-81,"group":-41,"loc":"3078.5 -1741"},

{"isGroup":true,"text":"","key":-82,"loc":"1201 -3061.75228125","dash":[2,4],"fill":"pink","color":"purple","group":-89},

{"isGroup":true,"text":"Dimensions of Opportunity","key":-83,"group":-82,"loc":"1201 -3053.089"},

{"text":"Diminished access to opportunities for achieving physical health and well-being: waiting for transport in unsafe neighborhood, missed medical appts.","key":-84,"loc":"1571 -3053.089","size":"180 100","color":"brown","group":-83},

{"text":"Diminished opportunities to expand mental and internal well-being through enrichment opportunities, museums, gyms, parks, etc","key":-85,"loc":"1381 -3053.089","size":"180 100","color":"brown","group":-83},

{"text":"Diminished opportunities to expand internal resources: leadership initiatives, enrichment activities, innovation and entrepreneurial spaces","key":-86,"loc":"1191 -3053.089","size":"180 100","color":"brown","group":-83},

{"text":"Diminished opportunities to expand community & social network; physical and mental growth & enrichment opportunities (e.g. museums, gyms, parks)","key":-87,"loc":"1001 -3053.089","size":"180 100","color":"brown","group":-83},

{"text":"Diminished opportunities to expand external resources: fresh food grocers, outdoor activities, parks, public spaces, etc","key":-88,"loc":"821 -3053.089","size":"160 100","color":"brown","group":-83},

{"isGroup":true,"text":"Housing","key":-89,"loc":"950 -3469.99978125","fill":"aliceblue"},

{"isGroup":true,"text":"Transportation","key":-90,"loc":"3308 -2250","fill":"beige"},

{"text":"Residential Segregation","key":-91,"loc":"-3056.375 -2314.817","size":"200 40","fill":"lightyellow","group":-157},

{"text":"Social Network Segregation [224[56]]","key":-92,"loc":"-3516.875 -2110.654","size":"200 40","fill":"lightyellow","group":-97},

{"text":"Barrier to education","key":-94,"loc":"-2926.375 -1864.817","size":"200 80","fill":"lightyellow","group":-157},

{"text":"Poor communities and minorities detached from valuable sources of employment information","key":-95,"loc":"-3196.875 -2110.654","size":"200 80","fill":"lightyellow","group":-97},

{"isGroup":true,"text":"","key":-97,"loc":"-3356.875 -2110.654","group":-101},

{"text":"Lack connections, referrals, and references needed to gain entry to good jobs and opportunities [224[57]]","key":-98,"loc":"-3356.875 -1970.654","size":"200 80","fill":"lightyellow","group":-101},

{"text":"People in disadvantaged communities have social networks with others from their communities who are also disadvantaged: social networks lack experienced mentors, influential job contacts, or persons with influence and power and decision-making authority","key":-99,"loc":"-3226.875 -1820.654","size":"200 140","fill":"lightyellow","group":-101},

{"text":"Across generations, advantaged communities tend to expand advantaged social networks; disadvantaged communities tend to expand disadvantaged networks","key":-100,"loc":"-3666.875 -1820.654","size":"200 100","fill":"white","group":-101,"color":"purple"},

{"isGroup":true,"text":"Social Marginalization","key":-101,"loc":"-3426.375 -1964.81728125","group":-157},

{"text":"High wage manufacturing jobs not located close to poor neighborhoods with urban minorities","key":-102,"loc":"-2573.875 -1567.3170000000002","size":"200 80","fill":"lightyellow","group":-115},

{"text":"Businesses locate to suburbs and locations desirable for their target market","key":-103,"loc":"-2573.875 -1657.317","size":"200 80","fill":"lightyellow","group":-115},

{"text":"SPATIAL BARRIERS: Spatial mismatch between where poor live and where jobs are located","key":-104,"loc":"-2313.875 -1617.317","size":"200 80","fill":"lightyellow","group":-115},

{"text":"Anti-Black Stereotypes","key":-106,"loc":"-2521.375 -2284.817","size":"200 40","fill":"lightyellow","group":-108},

{"text":"Residential Segregation","key":-107,"loc":"-2751.375 -2284.817","size":"200 40","fill":"lightyellow","group":-108},

{"isGroup":true,"text":"Economic Marginalization from Residential Segregation","key":-108,"loc":"-2636.375 -2284.817","group":-157},

{"text":"Neighborhood reputation & place discrimination + race discrimination: Subjective attitude towards a neighborhood as good/ bad, safe / dangerous depends significantly on its racial composition [225[59]]","key":-109,"loc":"-2558.875 -1982.317","size":"200 120","fill":"lightyellow","group":-115},

{"text":"Where to Locate Business: neighborhood reputation influences businesses decisions on location and employee recruitment [225[59]]","key":-110,"loc":"-2688.875 -1832.317","size":"200 100","fill":"lightyellow","group":-115},

{"text":"Who to Hire: employers make assumptions about competence, expertise, dependability, and commitment to work of potential employees based on residential location [225[59]]","key":-111,"loc":"-2438.875 -1832.317","size":"200 100","fill":"lightyellow","group":-115},

{"text":"Urban workers have difficulty finding and apply for these jobs; may not apply because of distance [224[55]]","key":-112,"loc":"-1843.875 -1592.3170000000002","size":"200 80","fill":"lightyellow","group":-115},

{"text":"Commuting costs are unaffordable for suburban jobs [224[55]]","key":-113,"loc":"-2073.875 -1642.317","size":"200 60","fill":"lightyellow","group":-115},

{"text":"Suburban job sites inaccessible via public transport [224[55]]","key":-114,"loc":"-2073.875 -1562.3170000000002","size":"200 60","fill":"lightyellow","group":-115},

{"isGroup":true,"text":"Barriers to Employment","key":-115,"loc":"-2266.375 -1784.817","group":-157},

{"text":"African Americans steered away from White neighborhoods during home search","key":-116,"loc":"-466.375 -2304.817","size":"200 80","fill":"lightyellow","group":-157},

{"text":"White Americans do not search for housing in predominantly Black neighborhoods","key":-117,"loc":"-926.375 -2294.817","size":"200 80","fill":"lightyellow","group":-157},

{"text":"Denied opportunities for quality housing, social mobility, and asset accumulation","key":-118,"loc":"-466.375 -2184.817","size":"200 80","fill":"lightyellow","group":-157},

{"text":"Reduced demand of housing in African American communities suppresses housing values; \"Segregation tax\" manifests as lower return on housing investment in African American community [225[61]]","key":-119,"loc":"-926.375 -2164.817","size":"260 80","fill":"lightyellow","group":-157},

{"text":"Less able than White Americans to benefit from appreciation of housing values, to build a reserve of wealth, and to break into the middle class, and to achieve upward mobility for themselves and their children [225[60]]","key":-120,"loc":"-648.875 -1829.817","size":"200 120","fill":"lightyellow","group":-132},

{"text":"African Americans less able than White Americans to use home ownership and equity as a vehicle to grow wealth; for every dollar earned and invested into home ownership, there is less return on investment; pipeline from hard work & human capital to investment to return is less advantageous for African American communities than White American communities ","key":-121,"loc":"-943.875 -1819.817","size":"320 120","fill":"lightyellow","group":-132},

{"text":"Racial wealth gap widen; racial wealth disparities are far greater than racial income disparities: Black families' median and mean wealth is less than 15 percent that of White families [FR]; being subject to or benefiting from barriers to housing, education, and labor markets can lead to lack of or accumulation of wealth that persists across generations [FR[9]]","key":-122,"loc":"-1296.375 -1484.8170000000002","size":"240 120","fill":"lightyellow","group":-157},

{"text":"African Americans and other minorities are less able to directly transfer wealth and resources across generations: less bequests & less inter vivos transfers (gifts) (e.g. down payment support to enable a home purchase or a substantial wedding gift) FR[(Charles and Hurst 2002)]. This can account for up to half of aggregate wealth [FR[8](Gale and Scholz 1994; Hamilton and Darrity 2010)]","key":-123,"loc":"-1116.375 -1664.817","size":"320 120","fill":"lightyellow","group":-157},

{"text":"African Americans and other minorities are less able to indirectly transfer wealth and resources across generations in the form of investments in their children that indirectly increase their wealth (e.g. investment in children's education by paying for college or private schools, which can increase the children's ability to accumulate wealth)","key":-124,"loc":"-1496.375 -1664.817","size":"320 120","fill":"lightyellow","group":-157},

{"text":"Deters wealth accumulation in current generation","key":-125,"loc":"-342.125 -1786.856","size":"320 40","fill":"lightyellow","group":-157},

{"text":"Deters wealth transfer across generations","key":-126,"loc":"-1352.125 -1776.856","size":"320 40","fill":"lightyellow","group":-157},

{"text":"Large percent of minorities & poor White families are asset poor [225[65]]","key":-128,"loc":"-486.375 -1684.817","size":"240 60","fill":"lightyellow","group":-157},

{"text":"Are just a few pay checks away from poverty and less able to protect children from downward mobility 225[66]]","key":-129,"loc":"-626.375 -1544.8170000000002","size":"240 60","fill":"lightyellow","group":-157},

{"text":"Have fragile personal safety net and are highly vulnerable to economic shocks (e.g. unemployment, medical emergencies); and are less able to cover expenses that can advantage next generation (e.g. college tuition); and less able to finance retirement [222]","key":-130,"loc":"-326.375 -1514.8170000000002","size":"240 120","fill":"lightyellow","group":-157},

{"text":"Discrimination in Property Insurance\n\n- Minorities more likely to pay higher premiums\n- Minorities more likely to be denied housing insurance required to qualify for a home loan [222[35]]\n","key":-127,"loc":"-3072.125 -2806.8559999999998","size":"200 160","fill":"lightyellow","group":-147},

{"text":"Gap in home values between Whites and minorities","key":-133,"loc":"-1516.375 -2004.817","size":"260 60","fill":"lightyellow","group":-157},

{"text":"Discriminatory appraisal of home value based on race (the same house with a White stand-in is appraised at a higher value compared to a Black stand-in)[NPR; NBC-Appr]","key":-134,"loc":"-1596.375 -2184.817","size":"200 100","fill":"lightyellow","group":-157,"thickness":4,"color":"blue"},

{"text":"Older age of entry into homeownership by African Americans (who tend to have less down-payment help from parents, as compared to White Americans with parental assistance and earlier age of entry) [FR[12]]","key":-135,"loc":"-1816.375 -2194.817","size":"200 120","fill":"lightyellow","group":-157},

{"text":"Gaps in purchase price and resource gaps [FR[12]]","key":-136,"loc":"-1396.375 -2184.817","size":"180 60","fill":"lightyellow","group":-157},

{"text":"Less housing appreciation in Black and minority neighborhoods [FR[12]]","key":-137,"loc":"-1196.375 -2184.817","size":"180 60","fill":"lightyellow","group":-157},

{"isGroup":true,"text":"","key":-132,"loc":"-826.375 -1824.817","group":-157},

{"text":"Discrimination by Lenders\n\n- African Americans & Hispanics receive less information, assistance, encouragement, and followup [221[32]]\n- less likely to be approved for housing loans or mortgage relief [221[33]]","key":-138,"loc":"-2802.125 -2806.8559999999998","size":"240 120","fill":"lightyellow","group":-147},

{"text":"Subprime mortgages: higher fees, higher interest rates [222[34]]","key":-139,"loc":"-2572.125 -2596.8559999999998","size":"240 60","fill":"lightyellow","group":-157},

{"text":"Predatory lending practices [222[34]]","key":-140,"loc":"-2572.125 -2526.8559999999998","size":"240 40","fill":"lightyellow","group":-157},

{"text":"Increased risk of foreclosure [222[34]]","key":-141,"loc":"-2222.125 -2586.8559999999998","size":"240 40","fill":"lightyellow","group":-157},

{"text":"Bad credit [222[34]]","key":-142,"loc":"-2222.125 -2526.8559999999998","size":"240 40","fill":"lightyellow","group":-157},

{"text":"Debilitating indebtedness [222[34]]","key":-143,"loc":"-2222.125 -2466.8559999999998","size":"240 40","fill":"lightyellow","group":-157},

{"text":"Discrimination by Real Estate Agents\n\n- withholding information about housing market availability\n- not permitting inspection of properties on market\n- offering less favorable terms and conditions\n- steering Whites to predominantly White neighborhoods and Blacks to predominantly minority neighborhoods [221[30,31]]","key":-144,"loc":"-3312.125 -2796.8559999999998","size":"200 220","fill":"lightyellow","group":-147},

{"text":"Economic valve and increasing spatial divide between rich and poor, widening since the 1970s: increasing spatial concentration of poverty and affluence because of creation of wealth for advantaged populations at the expense of other populations and the one-directional flow of advantage from one community to another","key":-145,"loc":"-636.375 -2034.817","size":"320 100","fill":"white","color":"purple","group":-157},

{"isGroup":true,"text":"Discriminatory Barriers Leading to Residential Segregation","key":-147,"loc":"-3047.125 -2796.8559999999998","group":-157},

{"text":"Restricted Housing Options","key":-146,"loc":"-1052.125 -2796.8559999999998","size":"240 40","fill":"lightyellow","group":-156},

{"text":"Housing discrimination limits wealth accumulation (homeownership is the largest investment by most families) [222[36]]","key":-148,"loc":"-742.125 -2786.8559999999998","size":"240 60","fill":"lightyellow","group":-156},

{"text":"Restricted opportunities based on neighborhoods of residence (\"where you live determines how long you live\")","key":-149,"loc":"-1052.125 -2686.8559999999998","size":"240 60","fill":"lightyellow","group":-156},

{"text":"Restricted educational opportunities","key":-150,"loc":"-1452.125 -2566.8559999999998","size":"240 60","fill":"lightyellow","group":-156},

{"text":"Restricted employment opportunities","key":-151,"loc":"-1162.125 -2566.8559999999998","size":"240 60","fill":"lightyellow","group":-156},

{"text":"Restricted social mobility","key":-152,"loc":"-902.125 -2566.8559999999998","size":"240 60","fill":"lightyellow","group":-156},

{"text":"Restricted options for safe and healthy residential environment","key":-153,"loc":"-622.125 -2566.8559999999998","size":"240 60","fill":"lightyellow","group":-156},

{"text":"Racial and ethnic minorities more likely to be confined to rental market [222[37]]","key":-154,"loc":"-472.125 -2816.8559999999998","size":"240 40","fill":"lightyellow","group":-156},

{"text":"If purchasing home, benefit less from appreciation in property values, leading to lower return per dollar invested in home [222[37]]","key":-155,"loc":"-472.125 -2746.8559999999998","size":"240 60","fill":"lightyellow","group":-156},

{"isGroup":true,"text":"","key":-156,"loc":"-962.125 -2686.8559999999998","group":-157},

{"isGroup":true,"text":"Residential Segregation and the Segregation of Opportunity","key":-157,"loc":"-1980 -2179.99978125","fill":"lightgreen"},

{"text":"High rent-to-income ratio","key":-158,"loc":"1347 -3590","size":"200 40","fill":"lightyellow","group":-68},

{"text":"insufficient money for other necessities (e.g. food, clothing, child care, transportation, heatlh care)","key":-159,"loc":"1347 -3510","size":"220 80","fill":"lightyellow","group":-68},

{"text":"Shelter poor: household cannot meet non-housing needs for some minimum level of adequacy after paying for housing [227[78,79]]","key":-160,"loc":"1347 -3410","size":"220 80","fill":"lightyellow","group":-68},

{"text":"Lower Boundary","key":-161,"loc":"1640 -480","size":"260 60","fill":"lightyellow"},

{"text":"Job openings appear on local searches","key":-162,"loc":"1600 -1330","size":"260 40","fill":"lightyellow","group":-184},

{"text":"Local businesses and internships may likely solicit applicants from local suburban schools and colleges ","key":-163,"loc":"1600 -1250","size":"260 60","fill":"lightyellow","group":-184},

{"text":"Rely on racially exclusive informal networks to recruit / connect applicants to jobs, internships, and other opportunities","key":-164,"loc":"1600 -1150","size":"260 60","fill":"lightyellow","group":-184},

{"text":"Recruitment strategies that exclude racial and ethnic minorities","key":-165,"loc":"1210 -1270","size":"260 100","fill":"lightyellow","group":-184},

{"text":"Unequal access to jobs; longer periods of unemployment; more likely to drop out of labor force; segregation into occupations with lower potential for socio-economic mobility, fewer opportunities for advancement, and lower earnings compared to White workers","key":-166,"loc":"1220 -1450","size":"260 120","fill":"lightyellow","group":-184},

{"text":"White sounding names (e.g. Greg, Nancy) get more interviews than Black sounding names (e.g. Jamal, Aisha) [220[14]]","key":-167,"loc":"1590 -1020","size":"260 60","fill":"lightyellow","color":"blue","thickness":4,"group":-184},

{"text":"Job applicants who are Black without a criminal record fare no better-and perhaps worse-than do whites with criminal records in getting initial consideration from employers [OJP]: being Black in the labor market is as much a liability as being an ex-offender","key":-168,"loc":"1590 -900","size":"260 120","fill":"lightyellow","color":"blue","thickness":4,"group":-184},

{"text":"People entering the job market from Black, minority [219[11]], and poor communities [QZA] more likely to be steered towards lower-paying jobs with minimal upward mobility","key":-169,"loc":"1590 -760","size":"260 80","fill":"lightyellow","color":"blue","thickness":4,"group":-184},

{"text":"Employer Discrimination","key":-170,"loc":"1220 -900","size":"260 80","fill":"lightyellow","color":"blue","thickness":4,"group":-184},

{"isGroup":true,"text":"Accruing Advantage for Advantaged Populations","key":-172,"group":-90,"loc":"4240 -1990"},

{"text":"Red-lining policies to decrease risk of insuring mortgages & loans","key":-173,"loc":"-2530 -3100","size":"240 60","fill":"lightgray"},

{"text":"Racist and discriminatory stereotypes about Blacks and other minorities","key":-174,"loc":"-140 -3230","size":"240 60","fill":"lightblue"},

{"text":"Jim Crow laws for segregation","key":-175,"loc":"-1310 -3410","size":"240 60","fill":"lightgray"},

{"text":"Blacks and Native Americans less than human in Constitution","key":-177,"loc":"-1960 -3590","size":"240 60","fill":"lightgray"},

{"text":"Blacks and Native Americans less than human in religious (cursed son of Noah) and social perceptions","key":-176,"loc":"-2070 -3520","size":"240 60","fill":"lightblue"},

{"text":"Slave trade from Africa","key":-178,"loc":"-1710 -3710","size":"240 60","fill":"lightgray"},

{"text":"Colonization of Native Nations","key":-179,"loc":"-1190 -3710","size":"240 60","fill":"lightgray"},

{"text":"Stripped off religion, language, names, identity, culture and forced to adopt new language, religion, names, identity; cultural genocide","key":-180,"loc":"-1560 -3580","size":"240 80","fill":"lightgray"},

{"text":"Acquisition of wealth through slave labor (e.g. cotton plantations)","key":-181,"loc":"-1710 -3860","size":"240 60","fill":"pink"},

{"text":"Acquisition of land as wealth through displacement, dispossession, and colonization of Native lands","key":-182,"loc":"-1340 -3860","size":"240 60","fill":"pink"},

{"text":"Enslavement of Native Nations","key":-183,"loc":"-1450 -3710","size":"240 60","fill":"lightgray"},

{"isGroup":true,"text":"Economic Exclusion","key":-184,"loc":"1405 -1115","fill":"lightgreen"},

{"text":"Accumulation of labor market disadvantages","key":-185,"loc":"2060 -1120","size":"260 60","fill":"lightyellow"},

{"text":"Lifetime earnings of racial and ethnic minorities less than of White workers [221[26]]","key":-186,"loc":"1920 -1300","size":"260 60","fill":"lightyellow"}

],

"linkDataArray": [

{"from":-9,"to":-12,"points":[3138.5,-2751,3138.5,-2741,3138.5,-2721,3133.5,-2721,3133.5,-2701,3133.5,-2691]},

{"from":-4,"to":-7,"points":[3918.5,-2621,3918.5,-2611,3918.5,-2591,3833.5,-2591,3833.5,-2571,3833.5,-2561]},

{"from":-14,"to":-8,"points":[3908.5,-2490,3908.5,-2480,3908.5,-2460.5,3908.5,-2460.5,3908.5,-2441,3908.5,-2431]},

{"from":-8,"to":-10,"points":[3908.5,-2351,3908.5,-2341,3908.5,-2201,3758.5,-2201,3608.5,-2201,3598.5,-2201]},

{"from":-11,"to":-10,"points":[3508.5,-2311,3508.5,-2301,3508.5,-2281,3508.5,-2281,3508.5,-2261,3508.5,-2251]},

{"from":-16,"to":-11,"points":[3508.5,-2480,3508.5,-2470,3508.5,-2455.5,3508.5,-2455.5,3508.5,-2441,3508.5,-2431]},

{"from":-17,"to":-15,"points":[3156.1,-2600,3156.1,-2590,3156.1,-2521,3239.8,-2521,3323.5,-2521,3333.5,-2521]},

{"from":-17,"to":-18,"points":[2999.3,-2600,2999.3,-2582,2999.3,-2571.5,2818.5,-2571.5,2818.5,-2561,2818.5,-2551]},

{"from":-17,"to":-19,"points":[2920.9000000000005,-2600,2920.9000000000005,-2590,2920.9000000000005,-2575.5,2568.5,-2575.5,2568.5,-2561,2568.5,-2551],"toSpot":"TopSide"},

{"from":-5,"to":-20,"points":[2268.5,-2621,2268.5,-2611,2268.5,-2586,2268.5,-2586,2268.5,-2561,2268.5,-2551]},

{"from":-20,"to":-22,"points":[2298.5,-2491,2298.5,-2481,2298.5,-2446,2348.5,-2446,2348.5,-2411,2348.5,-2401]},

{"from":-20,"to":-21,"points":[2238.5,-2491,2238.5,-2481,2238.5,-2446,2158.5,-2446,2158.5,-2411,2158.5,-2401]},

{"from":-22,"to":-23,"points":[2348.5,-2361,2348.5,-2351,2348.5,-2296,2301.833333333333,-2296,2301.833333333333,-2241,2301.833333333333,-2231]},

{"from":-21,"to":-23,"points":[2158.5,-2361,2158.5,-2351,2158.5,-2296,2215.166666666667,-2296,2215.166666666667,-2241,2215.166666666667,-2231]},

{"from":-23,"to":-24,"points":[2258.5,-2151,2258.5,-2141,2258.5,-2121,2258.5,-2121,2258.5,-2101,2258.5,-2091]},

{"from":-24,"to":-25,"points":[2258.5,-2011,2258.5,-2001,2258.5,-2001,2660,-2001,2660,-1911,2788.5,-1911,2798.5,-1911],"fromSpot":"BottomSide","toSpot":"LeftSide"},

{"from":-10,"to":-25,"points":[3508.5,-2151,3508.5,-2141,3508.5,-1911,3248.5,-1911,2988.5,-1911,2978.5,-1911],"fromSpot":"BottomSide","toSpot":"RightSide"},

{"from":-29,"to":-30,"points":[2918.5,-2320,2918.5,-2310,2918.5,-2275.5,2908.5,-2275.5,2908.5,-2241,2908.5,-2231]},

{"from":-30,"to":-31,"points":[2818.5,-2201,2808.5,-2201,2723.5,-2201,2723.5,-2201,2638.5,-2201,2628.5,-2201]},

{"from":-19,"to":-32,"points":[2568.5,-2491,2568.5,-2481,2568.5,-2461,2568.5,-2461,2568.5,-2441,2568.5,-2431]},

{"from":-32,"to":-31,"points":[2568.5,-2311,2568.5,-2301,2568.5,-2271,2568.5,-2271,2568.5,-2241,2568.5,-2231]},

{"from":-18,"to":-27,"points":[2818.5,-2491,2818.5,-2481,2818.5,-2451,2823.5,-2451,2823.5,-2421,2823.5,-2411]},

{"from":-33,"to":-34,"points":[2568.5,-2081,2568.5,-2071,2568.5,-2066,2568.5,-2066,2568.5,-2061,2568.5,-2051]},

{"from":-34,"to":-35,"points":[2568.5,-2011,2568.5,-2001,2568.5,-1990,2555.166666666667,-1990,2555.166666666667,-1979,2555.166666666667,-1961]},

{"from":-31,"to":-33,"points":[2568.5,-2171,2568.5,-2161,2568.5,-2156,2568.5,-2156,2568.5,-2151,2568.5,-2141],"toSpot":"TopSide","fromSpot":"BottomSide"},

{"from":-35,"to":-4,"points":[2648.5,-1911,2658.5,-1911,2660,-1911,2660,-1911,2668,-1911,2668,-2124,3236,-2124,3236,-2651,3848.5,-2651,3858.5,-2651],"toSpot":"LeftSide","dash":[4,4]},

{"from":-35,"to":-3,"points":[2601.833333333333,-1961,2601.833333333333,-1971,2601.833333333333,-1972,2668,-1972,2668,-2692,2913.5,-2692,2913.5,-2691,2913.5,-2681],"toSpot":"TopSide","dash":[4,4]},

{"from":-30,"to":-10,"points":[2998.5,-2201,3008.5,-2201,3208.5,-2201,3208.5,-2201,3408.5,-2201,3418.5,-2201]},

{"from":-23,"to":-31,"points":[2388.5,-2191,2398.5,-2191,2448.5,-2191,2448.5,-2201,2498.5,-2201,2508.5,-2201]},

{"from":-25,"to":-41,"points":[2888.5,-1861,2888.5,-1851,2888.5,-1845.66328125,2917.8333333333335,-1845.66328125,2917.8333333333335,-1840.3265625,2917.8333333333335,-
1830.3265625],"toSpot":"TopSide","fromSpot":"BottomSide"},

{"from":-41,"to":-42,"points":[3560.5,-1749.66328125,3570.5,-1749.66328125,3752,-1749.66328125,3752,-1730,3933.5,-1730,3943.5,-1730]},

{"from":-44,"to":-43,"points":[4103.5,-2120,4103.5,-2110,4103.5,-2049,4073.5,-2049,4073.5,-1988,4073.5,-1970]},

{"from":-45,"to":-44,"points":[4103.5,-2210,4103.5,-2200,4103.5,-2181,4103.5,-2181,4103.5,-2170,4103.5,-2160]},

{"from":-49,"to":-45,"points":[4514.5,-1820,4524.5,-1820,4524.5,-2240,4191.5,-2240,4191.5,-2250,4173.5,-2250],"fromSpot":"RightSide","toSpot":"RightSide"},

{"from":-45,"to":-48,"points":[4173.5,-2230,4183.5,-2230,4183.5,-2200,4423.5,-2200,4423.5,-1860,4423.5,-1850]},

{"from":-46,"to":-47,"points":[4293.5,-2020,4293.5,-2010,4293.5,-1935,4243.5,-1935,4243.5,-1860,4243.5,-1850]},

{"from":-42,"to":-43,"points":[4033.5,-1750,4033.5,-1760,4033.5,-1820,4103.5,-1820,4103.5,-1880,4103.5,-1890]},

{"from":-43,"to":-46,"points":[4133.5,-1970,4133.5,-1980,4133.5,-2060,4213.5,-2060,4213.5,-2060,4223.5,-2060],"toSpot":"LeftSide"},

{"from":-63,"to":-64,"points":[1117,-3545.584,1117,-3535.584,1117,-3530.584,1117,-3530.584,1117,-3525.584,1117,-3515.584]},

{"from":-62,"to":-63,"points":[1117,-3615.584,1117,-3605.584,1117,-3600.584,1117,-3600.584,1117,-3595.584,1117,-3585.584]},

{"from":-64,"to":-65,"points":[1117,-3475.584,1117,-3465.584,1117,-3460.584,1117,-3460.584,1117,-3455.584,1117,-3445.584]},

{"from":-59,"to":-67,"points":[1313.666666666667,-3715.584,1313.666666666667,-3697.584,1325.3333333333335,-3697.584,1325.3333333333335,-3700,1337,-3700,1337,-3690]},

{"from":-59,"to":-66,"points":[1380.3333333333333,-3715.584,1380.3333333333333,-3705.584,1380.3333333333333,-3705.584,1567,-3705.584,1567,-3685.584,1567,-3675.584]},

{"from":-57,"to":-62,"points":[1107,-3705.584,1107,-3695.584,1107,-3690.584,1117,-3690.584,1117,-3685.584,1117,-3675.584]},

{"from":-56,"to":-61,"points":[887,-3725.584,887,-3715.584,887,-3700.584,887,-3700.584,887,-3685.584,887,-3675.584]},

{"from":-68,"to":-69,"points":[1227,-3359,1227,-3349,1227,-3325.3759999999997,1211,-3325.3759999999997,1211,-3301.7519999999995,1211,-3291.7519999999995]},

{"from":-70,"to":-71,"points":[317,-3720,317,-3710,317,-3690,317,-3690,317,-3670,317,-3660]},

{"from":-71,"to":-72,"points":[317,-3560,317,-3550,317,-3530,317,-3530,317,-3510,317,-3500]},

{"from":-75,"to":-73,"points":[470.33333333333326,-3340,470.33333333333326,-3350,470.33333333333326,-3370.876,527.6666666666667,-3370.876,527.6666666666667,-3391.752,527.6666666666667,-3401.752]},

{"from":-75,"to":-72,"points":[403.6666666666665,-3340,403.6666666666665,-3350,403.6666666666665,-3370,317,-3370,317,-3390,317,-3400]},

{"from":-74,"to":-73,"points":[561,-3561.752,561,-3551.752,561,-3531.752,561,-3531.752,561,-3511.752,561,-3501.752]},

{"from":-76,"to":-74,"points":[551,-3721.752,551,-3711.752,551,-3691.752,561,-3691.752,561,-3671.752,561,-3661.752]},

{"from":-53,"to":-68,"points":[1011.6666666666667,-3879.584,1011.6666666666667,-3861.584,1011.6666666666667,-3829.084,1227,-3829.084,1227,-3796.584,1227,-3786.584]},

{"from":-77,"to":-41,"points":[3128.5,-1941,3128.5,-1931,3128.5,-1889.66328125,3239.1666666666665,-1889.66328125,3239.1666666666665,-1848.3265625,3239.1666666666665,-1830.3265625]},

{"from":-17,"to":-78,"points":[3077.7,-2600,3077.7,-2582,3077.7,-2580,3188.5,-2580,3188.5,-2132,3188.5,-2122]},

{"from":-78,"to":-77,"points":[3188.5,-2040,3188.5,-2030,3188.5,-2020.5,3128.5,-2020.5,3128.5,-2011,3128.5,-2001]},

{"from":-53,"to":-5,"points":[1162.3333333333333,-3879.584,1162.3333333333333,-3869.584,1162.3333333333333,-3868,2288.5,-3868,2288.5,-2699,2288.5,-2681],"dash":[4,4]},

{"from":-69,"to":-82,"points":[1211,-3211.7519999999995,1211,-3201.7519999999995,1211,-3177.08378125,1201,-3177.08378125,1201,-3152.4155625000003,1201,-3142.4155625000003]},

{"from":-91,"to":-92,"points":[-3106.375,-2294.817,-3106.375,-2284.817,-3106.375,-2212.7355,-3516.875,-2212.7355,-3516.875,-2140.654,-3516.875,-2130.654]},

{"from":-91,"to":-94,"points":[-3006.375,-2294.817,-3006.375,-2284.817,-3006.375,-2099.817,-2926.375,-2099.817,-2926.375,-1914.817,-2926.375,-1904.817]},

{"from":-91,"to":-95,"points":[-3056.375,-2294.817,-3056.375,-2276.817,-3056.375,-2218.7355,-3196.875,-2218.7355,-3196.875,-2160.654,-3196.875,-2150.654],"toSpot":"TopSide"},

{"from":-97,"to":-98,"points":[-3356.875,-2059.654,-3356.875,-2049.654,-3356.875,-2035.154,-3356.875,-2035.154,-3356.875,-2020.654,-3356.875,-2010.654]},

{"from":-99,"to":-100,"points":[-3326.875,-1820.654,-3336.875,-1820.654,-3446.875,-1820.654,-3446.875,-1820.654,-3556.875,-1820.654,-3566.875,-1820.654]},

{"from":-98,"to":-99,"points":[-3356.875,-1930.654,-3356.875,-1920.654,-3356.875,-1910.654,-3226.875,-1910.654,-3226.875,-1900.654,-3226.875,-1890.654]},

{"from":-103,"to":-104,"points":[-2473.875,-1657.317,-2463.875,-1657.317,-2443.875,-1657.317,-2443.875,-1630.6503333333333,-2423.875,-1630.6503333333333,-2413.875,-1630.6503333333333]},

{"from":-102,"to":-104,"points":[-2473.875,-1567.3170000000002,-2463.875,-1567.3170000000002,-2443.875,-1567.3170000000002,-2443.875,-1603.9836666666667,-2423.875,-1603.9836666666667,-2413.875,-
1603.9836666666667]},

{"from":-91,"to":-107,"points":[-2956.375,-2314.817,-2946.375,-2314.817,-2903.875,-2314.817,-2903.875,-2284.817,-2861.375,-2284.817,-2851.375,-2284.817],"dash":[4,4]},

{"from":-109,"to":-110,"points":[-2592.2083333333335,-1922.317,-2592.2083333333335,-1912.317,-2592.2083333333335,-1902.317,-2688.875,-1902.317,-2688.875,-1892.317,-2688.875,-1882.317]},

{"from":-109,"to":-111,"points":[-2525.5416666666665,-1922.317,-2525.5416666666665,-1912.317,-2525.5416666666665,-1902.317,-2438.875,-1902.317,-2438.875,-1892.317,-2438.875,-1882.317],"toSpot":"TopSide"},

{"from":-108,"to":-109,"points":[-2711.7083333333335,-2253.817,-2711.7083333333335,-2235.817,-2711.7083333333335,-2144.067,-2558.875,-2144.067,-2558.875,-2052.317,-2558.875,-2042.317]},

{"from":-114,"to":-112,"points":[-1973.875,-1562.3170000000002,-1963.875,-1562.3170000000002,-1958.875,-1562.3170000000002,-1958.875,-1578.983666666667,-1953.875,-1578.983666666667,-1943.875,-
1578.983666666667]},

{"from":-113,"to":-112,"points":[-1973.875,-1642.317,-1963.875,-1642.317,-1958.875,-1642.317,-1958.875,-1605.6503333333335,-1953.875,-1605.6503333333335,-1943.875,-1605.6503333333335]},

{"from":-104,"to":-113,"points":[-2213.875,-1630.6503333333333,-2203.875,-1630.6503333333333,-2193.875,-1630.6503333333333,-2193.875,-1642.317,-2183.875,-1642.317,-2173.875,-1642.317]},

{"from":-104,"to":-114,"points":[-2213.875,-1603.9836666666667,-2203.875,-1603.9836666666667,-2193.875,-1603.9836666666667,-2193.875,-1562.3170000000002,-2183.875,-1562.3170000000002,-2173.875,-
1562.3170000000002]},

{"from":-110,"to":-103,"points":[-2688.875,-1782.317,-2688.875,-1772.317,-2688.875,-1739.817,-2573.875,-1739.817,-2573.875,-1707.317,-2573.875,-1697.317]},

{"from":-116,"to":-118,"points":[-433.04166666666663,-2264.817,-433.04166666666663,-2246.817,-433.04166666666663,-2240.817,-466.375,-2240.817,-466.375,-2234.817,-466.375,-2224.817]},

{"from":-117,"to":-119,"points":[-926.375,-2254.817,-926.375,-2244.817,-926.375,-2233.817,-969.7083333333334,-2233.817,-969.7083333333334,-2222.817,-969.7083333333334,-2204.817]},

{"from":-108,"to":-116,"points":[-2410.375,-2295.1503333333335,-2400.375,-2295.1503333333335,-1487.375,-2295.1503333333335,-1487.375,-2354.817,-466.375,-2354.817,-466.375,-2344.817],"toSpot":"TopSide"},

{"from":-108,"to":-117,"points":[-2410.375,-2274.4836666666665,-2392.375,-2274.4836666666665,-1713.375,-2274.4836666666665,-1713.375,-2344.817,-926.375,-2344.817,-926.375,-2334.817],"toSpot":"TopSide"},

{"from":-124,"to":-122,"points":[-1496.375,-1604.817,-1496.375,-1594.817,-1496.375,-1574.817,-1336.375,-1574.817,-1336.375,-1554.8170000000002,-1336.375,-1544.8170000000002]},

{"from":-123,"to":-122,"points":[-1116.375,-1604.817,-1116.375,-1594.817,-1116.375,-1574.817,-1256.375,-1574.817,-1256.375,-1554.8170000000002,-1256.375,-1544.8170000000002]},

{"from":-126,"to":-124,"points":[-1405.4583333333333,-1756.856,-1405.4583333333333,-1746.856,-1405.4583333333333,-1740.8365,-1496.375,-1740.8365,-1496.375,-1734.817,-1496.375,-1724.817]},

{"from":-126,"to":-123,"points":[-1298.7916666666667,-1756.856,-1298.7916666666667,-1746.856,-1298.7916666666667,-1740.8365,-1116.375,-1740.8365,-1116.375,-1734.817,-1116.375,-1724.817]},

{"from":-125,"to":-128,"points":[-342.125,-1766.856,-342.125,-1756.856,-342.125,-1740.8365,-486.375,-1740.8365,-486.375,-1724.817,-486.375,-1714.817]},

{"from":-128,"to":-129,"points":[-526.375,-1654.817,-526.375,-1644.817,-526.375,-1614.817,-626.375,-1614.817,-626.375,-1584.8170000000002,-626.375,-1574.8170000000002]},

{"from":-128,"to":-130,"points":[-446.375,-1654.817,-446.375,-1644.817,-446.375,-1614.817,-326.375,-1614.817,-326.375,-1584.8170000000002,-326.375,-1574.8170000000002]},

{"from":-116,"to":-119,"points":[-499.7083333333333,-2264.817,-499.7083333333333,-2254.817,-499.7083333333333,-2234.817,-883.0416666666667,-2234.817,-883.0416666666667,-2214.817,-883.0416666666667,-
2204.817],"toSpot":"TopSide","fromSpot":"BottomSide"},

{"from":-134,"to":-133,"points":[-1596.375,-2134.817,-1596.375,-2124.817,-1596.375,-2092.817,-1516.375,-2092.817,-1516.375,-2060.817,-1516.375,-2034.817]},

{"from":-135,"to":-133,"points":[-1816.375,-2134.817,-1816.375,-2124.817,-1816.375,-2088.817,-1559.7083333333333,-2088.817,-1559.7083333333333,-2052.817,-1559.7083333333333,-2034.817],"fromSpot":"BottomSide"},

{"from":-136,"to":-133,"points":[-1396.375,-2154.817,-1396.375,-2144.817,-1396.375,-2098.817,-1473.0416666666667,-2098.817,-1473.0416666666667,-2052.817,-1473.0416666666667,-2034.817]},

{"from":-137,"to":-133,"points":[-1196.375,-2154.817,-1196.375,-2144.817,-1196.375,-2094.817,-1429.7083333333333,-2094.817,-1429.7083333333333,-2044.817,-1429.7083333333333,-2034.817]},

{"from":-108,"to":-133,"points":[-2561.0416666666665,-2253.817,-2561.0416666666665,-2243.817,-2561.0416666666665,-2244,-2561.0416666666665,-2244,-2561.0416666666665,-2124,-1603.0416666666667,-2124,-
1603.0416666666667,-2044.817,-1603.0416666666667,-2034.817],"toSpot":"TopSide"},

{"from":-118,"to":-132,"points":[-433.04166666666663,-2144.817,-433.04166666666663,-2126.817,-433.04166666666663,-2124,-433.04166666666663,-2124,-433.04166666666663,-1972,-730.2083333333334,-1972,-
730.2083333333334,-1910.817,-730.2083333333334,-1900.817]},

{"from":-119,"to":-132,"points":[-969.7083333333333,-2124.817,-969.7083333333333,-2106.817,-969.7083333333333,-2008.817,-922.5416666666667,-2008.817,-922.5416666666667,-1910.817,-922.5416666666667,-
1900.817]},

{"from":-133,"to":-132,"points":[-1516.375,-1974.817,-1516.375,-1964.817,-1516.375,-1850.1503333333333,-1320.625,-1850.1503333333333,-1124.875,-1850.1503333333333,-1114.875,-1850.1503333333333]},

{"from":-132,"to":-125,"points":[-537.875,-1799.4836666666667,-527.875,-1799.4836666666667,-520,-1799.4836666666667,-520,-1786.856,-512.125,-1786.856,-502.125,-1786.856]},

{"from":-132,"to":-126,"points":[-1114.875,-1799.4836666666667,-1124.875,-1799.4836666666667,-1153.5,-1799.4836666666667,-1153.5,-1776.856,-1182.125,-1776.856,-1192.125,-1776.856]},

{"from":-138,"to":-139,"points":[-2762.125,-2746.8559999999998,-2762.125,-2736.8559999999998,-2762.125,-2686.8559999999998,-2572.125,-2686.8559999999998,-2572.125,-2636.8559999999998,-2572.125,-
2626.8559999999998]},

{"from":-138,"to":-140,"points":[-2842.125,-2746.8559999999998,-2842.125,-2728.8559999999998,-2842.125,-2732,-2842.125,-2732,-2842.125,-2556,-2612.125,-2556,-2612.125,-2556.8559999999998,-2612.125,-
2546.8559999999998]},

{"from":-139,"to":-141,"points":[-2452.125,-2596.8559999999998,-2442.125,-2596.8559999999998,-2397.125,-2596.8559999999998,-2397.125,-2593.5226666666663,-2352.125,-2593.5226666666663,-2342.125,-
2593.5226666666663]},

{"from":-140,"to":-141,"points":[-2532.125,-2546.8559999999998,-2532.125,-2556.8559999999998,-2532.125,-2556.8559999999998,-2444,-2556.8559999999998,-2444,-2580.1893333333333,-2352.125,-2580.1893333333333,-
2342.125,-2580.1893333333333]},

{"from":-141,"to":-142,"points":[-2222.125,-2566.8559999999998,-2222.125,-2556.8559999999998,-2222.125,-2556.8559999999998,-2222.125,-2556.8559999999998,-2222.125,-2556.8559999999998,-2222.125,-
2546.8559999999998]},

{"from":-142,"to":-143,"points":[-2222.125,-2506.8559999999998,-2222.125,-2496.8559999999998,-2222.125,-2496.8559999999998,-2222.125,-2496.8559999999998,-2222.125,-2496.8559999999998,-2222.125,-
2486.8559999999998]},

{"from":-133,"to":-145,"points":[-1386.375,-2004.817,-1376.375,-2004.817,-1091.375,-2004.817,-1091.375,-2034.817,-806.375,-2034.817,-796.375,-2034.817]},

{"from":-118,"to":-145,"points":[-499.7083333333333,-2144.817,-499.7083333333333,-2134.817,-499.7083333333333,-2114.817,-583.0416666666667,-2114.817,-583.0416666666667,-2094.817,-583.0416666666667,-
2084.817]},

{"from":-119,"to":-145,"points":[-883.0416666666666,-2124.817,-883.0416666666666,-2114.817,-883.0416666666666,-2104.817,-689.7083333333334,-2104.817,-689.7083333333334,-2094.817,-689.7083333333334,-
2084.817],"toSpot":"TopSide","fromSpot":"BottomSide"},

{"from":-147,"to":-91,"points":[-3047.125,-2675.8559999999998,-3047.125,-2665.8559999999998,-3047.125,-2505.3365,-3056.375,-2505.3365,-3056.375,-2344.817,-3056.375,-2334.817]},

{"from":-146,"to":-148,"points":[-932.125,-2796.8559999999998,-922.125,-2796.8559999999998,-897.125,-2796.8559999999998,-897.125,-2786.8559999999998,-872.125,-2786.8559999999998,-862.125,-
2786.8559999999998]},

{"from":-146,"to":-149,"points":[-1052.125,-2776.8559999999998,-1052.125,-2766.8559999999998,-1052.125,-2746.8559999999998,-1052.125,-2746.8559999999998,-1052.125,-2726.8559999999998,-1052.125,-
2716.8559999999998]},

{"from":-149,"to":-150,"points":[-1124.125,-2656.8559999999998,-1124.125,-2646.8559999999998,-1124.125,-2626.8559999999998,-1452.125,-2626.8559999999998,-1452.125,-2606.8559999999998,-1452.125,-
2596.8559999999998]},

{"from":-149,"to":-151,"points":[-1076.125,-2656.8559999999998,-1076.125,-2638.8559999999998,-1076.125,-2622.8559999999998,-1162.125,-2622.8559999999998,-1162.125,-2606.8559999999998,-1162.125,-
2596.8559999999998]},

{"from":-149,"to":-152,"points":[-1028.125,-2656.8559999999998,-1028.125,-2638.8559999999998,-1028.125,-2622.8559999999998,-902.125,-2622.8559999999998,-902.125,-2606.8559999999998,-902.125,-
2596.8559999999998]},

{"from":-149,"to":-153,"points":[-980.125,-2656.8559999999998,-980.125,-2646.8559999999998,-980.125,-2626.8559999999998,-622.125,-2626.8559999999998,-622.125,-2606.8559999999998,-622.125,-
2596.8559999999998]},

{"from":-147,"to":-146,"points":[-2671.125,-2796.8559999999998,-2661.125,-2796.8559999999998,-1921.625,-2796.8559999999998,-1921.625,-2796.8559999999998,-1182.125,-2796.8559999999998,-1172.125,-
2796.8559999999998]},

{"from":-148,"to":-154,"points":[-622.125,-2796.8559999999998,-612.125,-2796.8559999999998,-607.125,-2796.8559999999998,-607.125,-2816.8559999999998,-602.125,-2816.8559999999998,-592.125,-
2816.8559999999998]},

{"from":-148,"to":-155,"points":[-622.125,-2776.8559999999998,-612.125,-2776.8559999999998,-607.125,-2776.8559999999998,-607.125,-2746.8559999999998,-602.125,-2746.8559999999998,-592.125,-
2746.8559999999998]},

{"from":-156,"to":-132,"points":[-341.125,-2633.1893333333333,-331.125,-2633.1893333333333,-331.125,-1850.1503333333333,-429.5,-1850.1503333333333,-527.875,-1850.1503333333333,-537.875,-
1850.1503333333333],"fromSpot":"RightSide","toSpot":"RightSide"},

{"from":-156,"to":-5,"points":[-341.125,-2740.5226666666663,-323.125,-2740.5226666666663,2248.5,-2740.5226666666663,2248.5,-2715.761333333333,2248.5,-2691,2248.5,-2681],"toSpot":"TopSide","dash":[4,4]},

{"from":-156,"to":-52,"points":[-962.125,-2847.8559999999998,-962.125,-2857.8559999999998,-962.125,-3940.584,119.9375,-3940.584,1202,-3940.584,1202,-3930.584],"toSpot":"TopSide","dash":[4,4]},

{"from":-53,"to":-73,"points":[861,-3910.584,851,-3910.584,760,-3910.584,760,-3391.752,561,-3391.752,561,-3401.752],"fromSpot":"LeftSide","toSpot":"BottomSide"},

{"from":-158,"to":-159,"points":[1347,-3570,1347,-3560,1347,-3560,1347,-3560,1347,-3560,1347,-3550]},

{"from":-159,"to":-160,"points":[1347,-3470,1347,-3460,1347,-3460,1347,-3460,1347,-3460,1347,-3450]},

{"from":-165,"to":-162,"points":[1340,-1295,1350,-1295,1405,-1295,1405,-1330,1460,-1330,1470,-1330]},

{"from":-165,"to":-163,"points":[1340,-1270,1358,-1270,1409,-1270,1409,-1250,1460,-1250,1470,-1250]},

{"from":-165,"to":-164,"points":[1340,-1245,1350,-1245,1350,-1245,1350,-1212,1600,-1212,1600,-1190,1600,-1180]},

{"from":-23,"to":-166,"points":[2215.1666666666665,-2151,2215.1666666666665,-2141,2215.1666666666665,-2140,1220,-2140,1220,-1520,1220,-1510]},

{"from":-115,"to":-166,"points":[-2266.375,-1516.3170000000002,-2266.375,-1506.3170000000002,-2266.375,-1508,-2266.375,-1508,-2266.375,-1412,-196,-1412,-196,-1450,1080,-1450,1090,-1450],"fromSpot":"BottomSide"},

{"from":-170,"to":-167,"points":[1220,-940,1220,-950,1220,-1020,1335,-1020,1450,-1020,1460,-1020]},

{"from":-170,"to":-168,"points":[1350,-900,1360,-900,1405,-900,1405,-900,1450,-900,1460,-900]},

{"from":-170,"to":-169,"points":[1220,-860,1220,-850,1220,-760,1335,-760,1450,-760,1460,-760]},

{"from":-172,"to":-9,"points":[4240,-2281,4240,-2291,4240,-2791,3734.25,-2791,3228.5,-2791,3218.5,-2791],"fromSpot":"TopSide","toSpot":"RightSide"},

{"from":-178,"to":-180,"points":[-1710,-3680,-1710,-3670,-1710,-3650,-1560,-3650,-1560,-3630,-1560,-3620]},

{"from":-179,"to":-180,"points":[-1190,-3680,-1190,-3670,-1190,-3580,-1310,-3580,-1430,-3580,-1440,-3580]},

{"from":-180,"to":-177,"points":[-1680,-3580,-1690,-3580,-1760,-3580,-1760,-3590,-1830,-3590,-1840,-3590]},

{"from":-181,"to":-178,"points":[-1710,-3830,-1710,-3820,-1710,-3785,-1710,-3785,-1710,-3750,-1710,-3740]},

{"from":-182,"to":-179,"points":[-1300,-3830,-1300,-3820,-1300,-3785,-1190,-3785,-1190,-3750,-1190,-3740]},

{"from":-182,"to":-183,"points":[-1380,-3830,-1380,-3820,-1380,-3785,-1450,-3785,-1450,-3750,-1450,-3740]},

{"from":-177,"to":-176,"points":[-1960,-3560,-1960,-3550,-2015,-3550,-2015,-3560,-2070,-3560,-2070,-3550]},

{"from":-176,"to":-175,"points":[-1950,-3520,-1940,-3520,-1690,-3520,-1690,-3410,-1440,-3410,-1430,-3410]},

{"from":-175,"to":-174,"points":[-1190,-3410,-1180,-3410,-725,-3410,-725,-3240,-270,-3240,-260,-3240]},

{"from":-174,"to":-173,"points":[-260,-3220,-270,-3220,-1335,-3220,-1335,-3100,-2400,-3100,-2410,-3100]},

{"from":-173,"to":-91,"points":[-2530,-3070,-2530,-3060,-2530,-2702.4085,-3023.0416666666665,-2702.4085,-3023.0416666666665,-2344.817,-3023.0416666666665,-2334.817]},

{"from":-174,"to":-134,"points":[-180,-3200,-180,-3190,-180,-3188,-1596.375,-3188,-1596.375,-2244.817,-1596.375,-2234.817]},

{"from":-174,"to":-170,"points":[-100,-3200,-100,-3190,-100,-900,490,-900,1080,-900,1090,-900],"toSpot":"LeftSide"},

{"from":-184,"to":-185,"points":[1741,-1115,1751,-1115,1835.5,-1115,1835.5,-1120,1920,-1120,1930,-1120]},

{"from":-185,"to":-186,"points":[2060,-1150,2060,-1160,2060,-1210,1920,-1210,1920,-1260,1920,-1270]},

{"from":-186,"to":-51,"points":[1920,-1330,1920,-1340,1920,-1340,1908,-1340,1908,-3700,1236,-3700,1236,-3772,972,-3772,972,-3880.584,972,-3890.584]},

{"from":-186,"to":-123,"points":[1790,-1300,1780,-1300,1780,-1300,1780,-1588,-620,-1588,-620,-1664.817,-946.375,-1664.817,-956.375,-1664.817]}

]}
